# Modeling Na_V_1.1/*SCN1A* sodium channel mutations in a microcircuit with realistic ion concentration dynamics suggests differential GABAergic mechanisms leading to hyperexcitability in epilepsy and hemiplegic migraine

**DOI:** 10.1371/journal.pcbi.1009239

**Published:** 2021-07-27

**Authors:** Louisiane Lemaire, Mathieu Desroches, Martin Krupa, Lara Pizzamiglio, Paolo Scalmani, Massimo Mantegazza

**Affiliations:** 1 Inria Sophia Antipolis Méditerranée Research Centre, MathNeuro Team, Valbonne-Sophia Antipolis, France; 2 Université Côte d’Azur, Nice, France; 3 Université Côte d’Azur, Laboratoire Jean-Alexandre Dieudonné, Nice, France; 4 Université Côte d’Azur, Institute of Molecular and Cellular Pharmacology (IPMC), Valbonne-Sophia Antipolis, France; 5 CNRS UMR7275, Institute of Molecular and Cellular Pharmacology (IPMC), Valbonne-Sophia Antipolis, France; 6 U.O. VII Clinical and Experimental Epileptology, Foundation IRCCS Neurological Institute Carlo Besta, Milan, Italy; 7 Inserm, Valbonne-Sophia Antipolis, France; Newcastle University, UNITED KINGDOM

## Abstract

Loss of function mutations of *SCN1A*, the gene coding for the voltage-gated sodium channel Na_V_1.1, cause different types of epilepsy, whereas gain of function mutations cause sporadic and familial hemiplegic migraine type 3 (FHM-3). However, it is not clear yet how these opposite effects can induce paroxysmal pathological activities involving neuronal networks’ hyperexcitability that are specific of epilepsy (seizures) or migraine (cortical spreading depolarization, CSD). To better understand differential mechanisms leading to the initiation of these pathological activities, we used a two-neuron conductance-based model of interconnected GABAergic and pyramidal glutamatergic neurons, in which we incorporated ionic concentration dynamics in both neurons. We modeled FHM-3 mutations by increasing the persistent sodium current in the interneuron and epileptogenic mutations by decreasing the sodium conductance in the interneuron. Therefore, we studied both FHM-3 and epileptogenic mutations within the same framework, modifying only two parameters. In our model, the key effect of gain of function FHM-3 mutations is ion fluxes modification at each action potential (in particular the larger activation of voltage-gated potassium channels induced by the Na_V_1.1 gain of function), and the resulting CSD-triggering extracellular potassium accumulation, which is not caused only by modifications of firing frequency. Loss of function epileptogenic mutations, on the other hand, increase GABAergic neurons’ susceptibility to depolarization block, without major modifications of firing frequency before it. Our modeling results connect qualitatively to experimental data: potassium accumulation in the case of FHM-3 mutations and facilitated depolarization block of the GABAergic neuron in the case of epileptogenic mutations. Both these effects can lead to pyramidal neuron hyperexcitability, inducing in the migraine condition depolarization block of both the GABAergic and the pyramidal neuron. Overall, our findings suggest different mechanisms of network hyperexcitability for migraine and epileptogenic Na_V_1.1 mutations, implying that the modifications of firing frequency may not be the only relevant pathological mechanism.

## Author summary

The voltage-gated sodium channel Na_V_1.1 is a major target of human mutations implicated in different pathologies. In particular, mutations identified in certain types of epilepsy cause loss of function of the channel, whereas mutations identified in certain types of migraine (in which spreading depolarizations of the cortical circuits of the brain are involved) cause instead gain of function. Here, we study dysfunctions induced by these differential effects in a two-neuron (GABAergic and pyramidal) conductance-based model with dynamic ion concentrations. We obtain results that can be related to experimental findings in both situations. Namely, extracellular potassium accumulation induced by the activity of the GABAergic neuron in the case of CSD, and higher propensity of the GABAergic neuron to depolarization block in the epileptogenic scenario, without significant modifications of its firing frequency prior to it. Both scenarios can induce hyperexcitability of the pyramidal neuron, leading in the migraine condition to depolarization block of both the GABAergic and the pyramidal neuron. Our results are successfully confronted to experimental data and suggest that modification of firing frequency is not the only key mechanism in these pathologies of neuronal excitability.

## 1 Introduction

Na_V_1.1 is a voltage-gated sodium channel mainly expressed in GABAergic neurons and it is crucial for their excitability. Mutations of *SCN1A*, the gene coding for this channel, cause either sporadic/familial hemiplegic migraine (FHM) or epilepsy [1–3].

FHM is a rare but severe subtype of migraine with aura, which typically includes hemiparesis, i.e. weakness of one side of the body. Three responsible genes for FHM are currently known. *SCN1A* was the last of them to be identified [4], causing FHM type 3 (FHM-3). Although one study initially reported loss of function for two FHM-3 Na_V_1.1 mutations studied in heterologous expression systems [5], more recent works have instead established gain of function of the channel as the common functional effect of FHM-3 mutations [2, 3, 6–9]. A pathological mechanism of migraine with aura is cortical spreading depolarization (CSD), a wave of transient intense neuronal firing followed by a sustained depolarization block, accompanied by breakdown of the transmembrane ion concentration gradients, water influx and cell swelling, which slowly propagates in the cortex [10–13]. Numerous clinical studies have shown that spreading depolarizations are involved in different neurological diseases, including cerebral ischemia and traumatic brain injury [11, 13, 14]. Common features of all spreading depolarizations are the magnitude of the neuronal depolarization, the changes in ion gradients involved, the water influx with neuronal swelling, the waveform of the negative direct current (DC) shift, the changes in holding current and input resistance of patch-clamped neurons, the intrinsic optical signal changes, and the abrupt release of neurotransmitters, including both excitatory neurotransmitters such as glutamate and inhibitory neurotransmitters such as GABA [11, 15]. Although there are numerous studies performed with experimental animals [11, 16], clinical evidence linking CSD and migraine symptoms is more limited. However, a recent report has unequivocally demonstrated with electrophysiological recordings that spreading depolarization-induced spreading depression of spontaneous cortical activity was linked to symptomatic migraine aura in a patient [17]. In addition to the generation of the patient percept of migraine aura, it has been proposed that in migraineurs spreading depolarizations can stimulate trigeminal nociceptors innervating the meninges, activating pain pathways and provoking the headache [11, 13, 18]. *In vivo* experiments on the knock-in *Scn1a*^L263V/+^ mouse model have shown increased propensity to CSD in this model and suggest that FHM-3 mutations predominantly affect CSD initiation, rather than its propagation, because no increase of CSD propagation speed was observed in this model [19], unlike what was reported for the two other types of FHM (FHM-1 and FHM-2) [20, 21]. However, the link between FHM-3 mutations and the initiation of CSD is not well understood yet. In particular, it is unclear how gain of function mutations in voltage-gated sodium channels of GABAergic neurons, which classically have an inhibitory role, can lead to the network hyperexcitability characterizing CSD. Studying this link can give a better understanding not only of FHM-3 pathophysiology, but also of CSD and migraine aura in general, as well as stroke, traumatic brain injury and other pathologies in which spreading depolarizations are involved.

On the other hand, mutations of the same gene have been found in patients with epileptic disorders. This is the case of Dravet syndrome [22], a severe and pharmacoresistant developmental and epileptic encephalopathy, and of genetic epilepsy with febrile seizures plus (GEFS+) [23], characterized in general by milder phenotypes. These Na_V_1.1 epileptogenic mutations cause a loss of function of the channel [1–3, 24, 25]. The causal link between the loss of function of Na_V_1.1 and epileptiform activity is certainly less counterintuitive than the one between gain of function and CSD. Indeed, it is expected that a failure of excitability in inhibitory neurons can promote seizures. However, here also there is no consensus when it comes to the precise mechanisms of seizure generation involved. It is important to investigate how mutations with opposite effects lead to one or the other of two different manifestations of neuronal hyperactivity: CSD and epileptic seizures [12].

The present study does not aim to model a full blown CSD or a seizure. Instead, we focused on their initiation and on the conditions which can lead to it. We used a modeling approach, building upon previous work. In [26], we developed a two-neuron (GABAergic and pyramidal) conductance-based model, which partially accounted for ion concentration dynamics. We assumed that FHM-3 mutations cause hyperactivity of the GABAergic neuron, and found that it promotes CSD initiation in the model. The simulations highlighted the key role of spiking-induced extracellular potassium build-up. Here, we substantially improved the model from [26]: we explicitly modeled FHM-3 mutations, added the implementation of epileptogenic mutations, and modeled ion concentration dynamics more consistently. A more detailed description of the modifications compared to [26] is given in Section 2.2. Several other modeling studies have addressed issues similar to those of interest here, using conductance-based models with dynamic ion concentrations. For instance, Florence et al. [27] suggested that extracellular potassium is fundamental for epileptiform bursting and spreading depolarization [27]. Wei et al. proposed a unified model for studying those two pathological behaviors [28]. Dahlem et al. modeled FHM-3 mutations and concluded that they render gray matter tissue more vulnerable to spreading depolarization. However, the main novelty of our approach is that we implemented Na_V_1.1 mutations on a GABAergic neuron, and analyzed their effects on a microcircuit formed by the GABAergic neuron and an interconnected pyramidal neuron. This allowed us to take into account the inhibitory effect of GABAergic neurons on pyramidal neurons. We studied both FHM-3 and epileptogenic mutations within the same framework, modifying only two relevant parameter values. We present original experimental results that support predictions of the model and we put the model simulations into perspective with other experimental works. In particular, we qualitatively compared them with results we obtained using the Hm1a Na_V_1.1 enhancer to mimic FHM-3 mutations [29] and with results of Freilinger et al. (personal communication; see acknowledgments), who generated the knock-in mouse model of the L1649Q FHM-3 mutation studying effects on microcircuit features.

## 2 Materials and methods

### 2.1 Ethics statement

Experiments with mice were carried out according to the European directive 2010/63/UE and approved by institutional and ethical committees (PEA216-04551.02, France; 711/2016-PR, Italy). All efforts were made to minimize the number of animals used and their suffering. Animals were group housed (5 mice per cage, or 1 male and 2 females per cage for breeding) on a 12 h light/dark cycle, with water and food ad libitum.

### 2.2 The model

**Fig 1:**
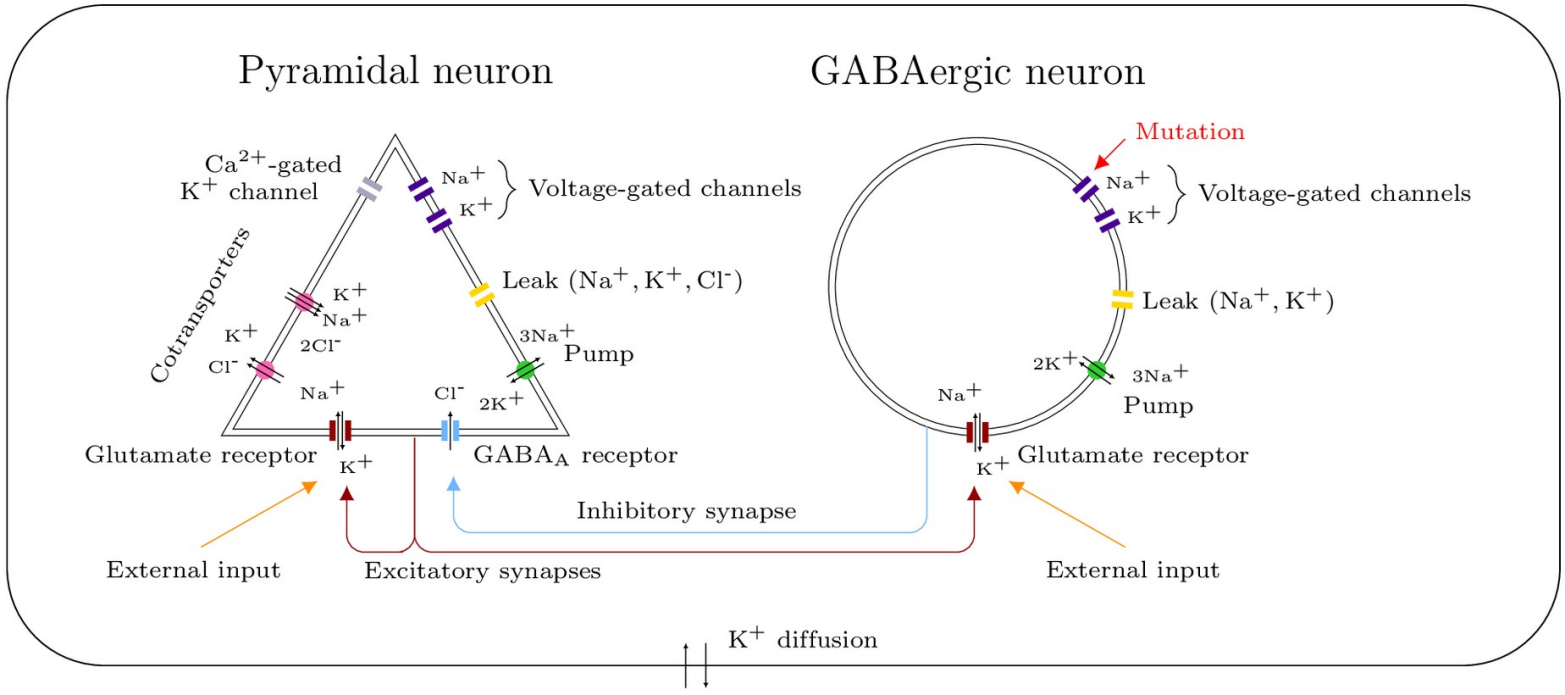
Schematic representation of the model. Consider a pair of interconnected neurons in a closed volume. We modeled a GABAergic synapse from the GABAergic neuron to the pyramidal one, a glutamatergic synapse from the pyramidal neuron to the GABAergic one and a glutamatergic autapse from the pyramidal neuron to itself. The ion transport mechanisms represented here generate transmembrane ionic currents, which modify the membrane potentials of the neurons and the ion concentrations in the different compartments. The diffusion of extracellular potassium takes into account both passive diffusion and glial buffering. We modeled external stimuli, which reflect the activity of the surrounding network or mimic experimental depolarizations, with glutamate inputs on the glutamatergic receptors. The implementation of Na_V_1.1’s genetic mutations affects only the GABAergic neuron.

We developed a conductance-based model formed by a pair of neurons: a GABAergic interneuron and a glutamatergic pyramidal neuron. This model takes into account the dynamics of ion concentrations. It is an essential feature here, since ion gradients, and hence reversal potentials, are modified during migraine and epilepsy attacks. The two neurons are thus coupled through variations of extracellular ion concentrations, in addition to synaptic connections. We implemented several ion transport proteins, such as voltage-gated channels, cotransporters, pumps and synaptic channels, which are sketched in Fig 1. The dynamics of the state variables is given by a system of 18 differential equations: system (1). The variables are listed in Table 1, the parameters and their default values in Table 2. We performed numerical simulations of the model with the software package XPPAUT [30], using a 4th-order Runge-Kutta scheme. The code is available in ModelDB [31] at http://modeldb.yale.edu/267047.

$$C\;\frac{dv_{e}}{dt}=-I_{\text{Na},e}-I_{K,e}-I_{\text{Cl},e}$$ (1a)

$$\frac{dm_{e}}{dt}=\alpha_{m,e}\left(1-m_{e}\right)-\beta_{m,e}\;m_{e}$$ (1b)

$$\frac{dh_{e}}{dt}=\alpha_{h,e}\left(1-h_{e}\right)-\beta_{h,e}\;h_{e}$$ (1c)

$$\frac{dn_{e}}{dt}=\alpha_{n,e}\left(1-n_{e}\right)-\beta_{n,e}\;n_{e}$$ (1d)

$$\frac{d{\left[{\text{K}}^{+}\right]}_{e}}{dt}=-\gamma_{e}\;I_{K,e}$$ (1e)

$$\frac{d{\left[{\text{Na}}^{+}\right]}_{e}}{dt}=-\gamma_{e}\;I_{\text{Na},e}$$ (1f)

$$\frac{d{\left[{\text{Cl}}^{-}\right]}_{e}}{dt}=\gamma_{e}\;I_{\text{Cl},e}$$ (1g)

$$\frac{d{\left[{\text{Ca}}^{2+}\right]}_{e}}{dt}=-\frac{\gamma_{e}}{2}\;I_{\text{Ca},e}-\frac{{\left[{\text{Ca}}^{2+}\right]}_{e}}{\tau_{\text{Ca}}}$$ (1h)

$$\frac{ds_{e}}{dt}=-\frac{1}{\tau_{e}}s_{e}\;,\;\text{if}\;v_{e}=v_{\text{thres},e}\;\;\text{and}\;\;\frac{{\text{dv}}_{e}}{dt}>0\;\text{then}\;s_{e}\leftarrow 1$$ (1i)

$$C\;\frac{dv_{i}}{dt}=-I_{\text{Na},i}-I_{K,i}$$ (1j)

$$\frac{dh_{i}}{dt}=\frac{h_{\infty ,i}-h_{i}}{\tau_{h,i}}$$ (1k)

$$\frac{dn_{i}}{dt}=\frac{n_{\infty ,i}-n_{i}}{\tau_{n,i}}$$ (1l)

$$\frac{d{\left[{\text{K}}^{+}\right]}_{i}}{dt}=-\gamma_{i}\;I_{K,i}$$ (1m)

$$\frac{d{\left[{\text{Na}}^{+}\right]}_{i}}{dt}=-\gamma_{i}\;I_{\text{Na},i}$$ (1n)

$$\frac{ds_{i}}{dt}=-\frac{1}{\tau_{i}}s_{i}\;,\;\text{if}\;v_{i}=v_{\text{thres},i}\;\;\text{and}\;\;\frac{{\text{dv}}_{i}}{dt}>0\;\text{then}\;s_{i}\leftarrow 1$$ (1o)

$$\frac{d{\left[{\text{K}}^{+}\right]}_{o}}{dt}=\frac{{\text{Vol}}_{e}}{{\text{Vol}}_{o}}\gamma_{e}\;I_{K,e}+\frac{{\text{Vol}}_{i}}{{\text{Vol}}_{o}}\gamma_{i}\;I_{K,i}-I_{K,\text{diff}}$$ (1p)

$$\frac{d{\left[{\text{Na}}^{+}\right]}_{o}}{dt}=\frac{{\text{Vol}}_{e}}{{\text{Vol}}_{o}}\gamma_{e}\;I_{\text{Na},e}+\frac{{\text{Vol}}_{i}}{{\text{Vol}}_{o}}\gamma_{i}\;I_{\text{Na},i}$$ (1q)

$$\frac{d{\left[{\text{Cl}}^{-}\right]}_{o}}{dt}=-\frac{{\text{Vol}}_{e}}{{\text{Vol}}_{o}}\gamma_{e}\;I_{\text{Cl},e}$$ (1r)

**Table 1:** Variables of system (1).

Variable	Description	Unit
*t*	Time	ms
	**Pyramidal neuron**	
*v*_e_	Membrane potential	mV
*m*_e_	Sodium activating gating variable	
*h*_e_	Sodium inactivating gating variable	
*n*_e_	Potassium activating gating variable	
[K^+^]_e_	Intracellular potassium concentration	mM
[Na^+^]_e_	Intracellular sodium concentration	mM
[Ca^−^]_e_	Intracellular chloride concentration	mM
[Ca^2+^]_e_	Intracellular calcium concentration	mM
*s*_e_	Synaptic variable	
	**GABAergic neuron**	
*v*_i_	Membrane potential	mV
*h*_i_	Sodium inactivating gating variable	
*n*_i_	Potassium activating gating variable	
[K^+^]_i_	Intracellular potassium concentration	mM
[Na^+^]_i_	Intracellular sodium concentration	mM
*s*_i_	Synaptic variable	
	**Extracellular concentrations**	
[K^+^]_o_	Extracellular potassium concentration	mM
[Na^+^]_o_	Extracellular sodium concentration	mM
[Cl^−^]_o_	Extracellular chloride concentration	mM

**Table 2:** Model parameters.

Parameter	Description	Value	Unit	Source
*C*	Membrane capacitance per area unit	1	μF ⋅ cm^−2^	[26]
*β*_2_	Ratio GABAergic over pyramidal neuron volume	$\frac{2}{3}$		-
Vol_e_	Pyramidal neuron volume	1.4368 ⋅ 10^−9^	cm^3^	[26]
*T*	Temperature	309.15	K	[26]
*ρ*_pump,−70_	Pump maximal rate at −70 mV	30	μA ⋅ cm^−2^	-
*K*_pump,Na_	Pump half activation intracellular [Na^+^]	7.7	mM	[35]
*K*_pump,K_	Pump half activation extracellular [K^+^]	2	mM	[35]
*a*	Parameter for the pump voltage dependence	0.39		[36]
*b*	Parameter for the pump voltage dependence	1.28		[36]
*ε*	Extracellular K^+^ diffusion rate	5 ⋅ 10^−4^	ms^−1^	-
*K*_bath_	K^+^ bath concentration	3.5	mM	[26, 28]
	**Pyramidal neuron**			
*τ*_e_	Time constant for the decay of *s*_e_	3	ms	[26, 37]
*v*_thres,e_	Voltage threshold defining firing time	0	mV	[26, 38]
*g*_Na,FI,e_	Fast inactivating Na^+^ maximal conductance	100	mS ⋅ cm^−2^	[26, 39]
*g*_K,DR,e_	Delayed rectifier K^+^ maximal conductance	80	mS ⋅ cm^−2^	[26, 39]
*g*_K,AHP,e_	Ca^2+^-activated K^+^ maximal conductance	1	mS ⋅ cm^−2^	-
*K*_Ca_	Ca^2+^-activated K^+^ half activation [Ca^2+^]_e_	0.001	mM	[26, 40]
*g*_Na,L,e_	Na^+^ leak conductance	0.015	mS ⋅ cm^−2^	-
*g*_K,L,e_	K^+^ leak conductance	0.05	mS ⋅ cm^−2^	[26]
*g*_Cl,L,e_	Cl^−^ leak conductance	0.015	mS ⋅ cm^−2^	[26]
*ρ*_KCC_	KCC2 cotransporter strength	0.0003	mM ⋅ ms^−1^	[26, 28]
*ρ*_NKCC_	NKCC1 cotransporter strength	0.0001	mM ⋅ ms^−1^	[26, 28]
*K*_NKCC,K_	NKCC1 cotransporter half activation [K^+^]_o_	16	mM	[26, 28]
*g*_GLU,e_	Glutamatergic current maximal conductance	0.1	mS ⋅ cm^−2^	[26]
*g*_GABA,e_	GABAergic current maximal conductance	2.5	mS ⋅ cm^−2^	-
*g*_D,e_	External glutamatergic conductance	0-0.3	mS ⋅ cm^−2^	-
*g*_Ca,e_	Ca^2+^ maximal conductance	1	mS ⋅ cm^−2^	[26, 40]
*E*_Ca,e_	Ca^2+^ reversal potential	120	mV	[26, 40]
*τ*_Ca_	Time constant for Ca^2+^ extrusion and buffering	80	ms	[26, 41]
	**GABAergic neuron**			
*τ*_i_	Time constant for the decay of *s*_i_	9	ms	[26]
*v*_thres,i_	Voltage threshold defining firing time	0	mV	[26, 38]
*g*_Na,FI,i_	Fast inactivating Na^+^ maximal conductance	112.5	mS ⋅ cm^−2^	[33]
*g*_K,DR,i_	Delayed rectifier K^+^ maximal conductance	225	mS ⋅ cm^−2^	[33]
*g*_Na,L,i_	Na^+^ leak conductance	0.012	mS ⋅ cm^−2^	-
*g*_K,L,i_	K^+^ leak conductance	0.05	mS ⋅ cm^−2^	-
*g*_GLU,i_	Glutamatergic current maximal conductance	0.1	mS ⋅ cm^−2^	[26]
*g*_D,i_	External glutamatergic conductance	0-0.3	mS ⋅ cm^−2^	-

This model is based upon previous work [26], to which we made the following key improvements:

1. We modeled Na_V_1.1’s FHM-3 and epileptogenic mutations, considering their effect on the GABAergic neuron. The implementation of those mutations is detailed in Section 2.2.6.
2. We propose a more consistent modeling of ion concentration dynamics. In [26], the reversal potentials of the GABAergic neuron were assumed to be constant, and only part of the ion currents of the pyramidal neuron had an effect on its reversal potentials, in addition to the delayed-rectifier potassium current of the GABAergic neuron. Here, we took into account the effect of each transmembrane current on the intracellular ion concentration of the corresponding neuron and on the extracellular concentration. As a consequence, system (1) has first integrals linking the membrane potential of a neuron and its intracellular ion concentrations, as we show in Section 2.2.1.
3. In [26], the dynamics of the voltage-gated channels of the GABAergic neuron was described using the Wang-Buzsáki model of hippocampal interneurons [32]. We replaced it with a model of fast-spiking cortical interneurons by Golomb et al. [33], which is presented in Section 2.2.5. Indeed, CSD that causes migraine aura is generated in the neocortex and experimental results suggest the FHM-3 CSD is selectively initiated in the neocortex [29].
4. We included the activity of the Na^+^/K^+^ ATPase for both neurons, not only for the pyramidal one as in [26]. We replaced the expression describing its dependence on the intracellular sodium and extracellular potassium concentrations with a more realistic one developed by Kager et al. [34], which is based on experimental data.

#### 2.2.1 Conserved quantities

We identified several first integrals in system (1), namely conservation of mass for sodium and chloride, and a relationship between the membrane potential of a neuron and its intracellular concentrations. In this section, we will explain their role and effect on the system’s dynamics. First of all, we introduce the conversion factors *γ*_e_, *γ*_i_, *β*_1_ and *β*_2_.

**Conversion factors**. Let *z* be the valence of an ion. Then, $\frac{\gamma_{e}}{z}$ converts the current of this ion across the membrane of the pyramidal neuron in μA ⋅ cm^−2^ to variation of concentration inside the cell in mM ⋅ ms^−1^. The conversion factor *γ*_e_ is defined as

$$\begin{array}{c} \gamma_{e}=\frac{S_{e}}{10^{3}{\text{Vol}}_{e}N_{A}e}, \end{array}$$

where Vol_e_ and *S*_e_ are the volume and surface area of the pyramidal neuron, *e* = 1.6 ⋅ 10^−19^ C the elementary charge and *N*_A_ = 6.02 * 10^23^ mol^−1^ the Avogadro number. We take Vol_e_ = 1.4368 * 10^−9^ cm^3^ [26] and we compute *S*_e_ assuming the neuron is spherical:

$$\begin{array}{c} S_{e}=4\pi (\frac{3{\text{Vol}}_{e}}{4\pi }{)}^{\frac{2}{3}}. \end{array}$$

We obtain

$$\begin{array}{c} \gamma_{e}=4.45*10^{-5}\text{mol}\cdot {\text{cm}}^{2}\cdot \mu {\text{C}}^{-1}\cdot {\text{L}}^{-1}. \end{array}$$

The conversion factor *γ*_i_ plays the same role as *γ*_e_ but for the GABAergic neuron. We assume that the GABAergic neuron is a sphere of volume Vol_i_ = *β*_2_Vol_e_, with $\beta_{2}=\frac{2}{3}$, which gives

$$\begin{array}{c} \gamma_{i}=5.09*10^{-5}\text{mol}\cdot {\text{cm}}^{2}\cdot \mu {\text{C}}^{-1}\cdot {\text{L}}^{-1}. \end{array}$$

To convert variation of intracellular concentration to variation of extracellular concentration, we need to multiply by the intracellular volume of the corresponding neuron and to divide by the extracellular volume Vol_o_. Let *β*_1_ be the ratio of total intracellular volume over extracellular volume: $\beta_{1}=\frac{{\text{Vol}}_{e}+{\text{Vol}}_{i}}{{\text{Vol}}_{o}}=4$ [42]. We then have

$$\begin{array}{cc} \frac{{\text{Vol}}_{e}}{{\text{Vol}}_{o}} & =\frac{\beta_{1}}{1+\beta_{2}},\\ \frac{{\text{Vol}}_{i}}{{\text{Vol}}_{o}} & =\frac{\beta_{1}\beta_{2}}{1+\beta_{2}}. \end{array}$$

**Conservation of mass**. In system (1), the sodium and chloride concentrations are only modified by transmembrane currents. We have thus conservation of mass for those ions. Note that it is not the case for potassium, for which we take into account extracellular diffusion (see Section 3.1.4). The conserved quantities enable us to reduce the number of unknowns. For sodium,

$$\begin{array}{c} \frac{d}{dt}({\left[{\text{Na}}^{+}\right]}_{o}{\text{Vol}}_{o}+{\left[{\text{Na}}^{+}\right]}_{e}{\text{Vol}}_{e}+{\left[{\text{Na}}^{+}\right]}_{i}{\text{Vol}}_{i})=0. \end{array}$$

Let

$$\begin{array}{c} {\text{Na}}_{\sum }={\left[{\text{Na}}^{+}\right]}_{o}+\frac{{\text{Vol}}_{e}}{{\text{Vol}}_{o}}{\left[{\text{Na}}^{+}\right]}_{e}+\frac{{\text{Vol}}_{i}}{{\text{Vol}}_{o}}{\left[{\text{Na}}^{+}\right]}_{i}. \end{array}$$

We fix

$$\begin{array}{c} {\text{Na}}_{\sum }=145+\beta_{1}10=185\;\text{mM}. \end{array}$$

We can then express the extracellular sodium concentration as a function of the intracellular concentrations:

$$\begin{array}{c} {\left[{\text{Na}}^{+}\right]}_{o}={\text{Na}}_{\sum }-\frac{{\text{Vol}}_{e}}{{\text{Vol}}_{o}}{\left[{\text{Na}}^{+}\right]}_{e}-\frac{{\text{Vol}}_{i}}{{\text{Vol}}_{o}}{\left[{\text{Na}}^{+}\right]}_{i}. \end{array}$$

Similarly, for chloride we define

$$\begin{array}{c} {\text{Cl}}_{\sum }={\left[{\text{Cl}}^{-}\right]}_{o}+\frac{{\text{Vol}}_{e}}{{\text{Vol}}_{o}}{\left[{\text{Cl}}^{-}\right]}_{e} \end{array}$$

and choose

$$\begin{array}{c} {\text{Cl}}_{\sum }=130+\frac{\beta_{1}}{1+\beta_{2}}5=142\;\text{mM}. \end{array}$$

**Relation between membrane potential and intracellular ion concentrations**. System (1) has two other first integrals, which arise from the fact that we take into account the effect of all ionic currents on the dynamics of the intracellular concentrations and that no other mechanism affects those concentrations. For the pyramidal neuron, we have

$$\begin{array}{c} C\frac{dv_{e}}{dt}-\frac{1}{\gamma_{e}}(\frac{d{\left[{\text{Na}}^{+}\right]}_{e}}{dt}+\frac{d{\left[{\text{K}}^{+}\right]}_{e}}{dt}-\frac{d{\left[{\text{Cl}}^{-}\right]}_{e}}{dt})=0. \end{array}$$ (2)

This allows us to remove an additional unknown. Let

$$\begin{array}{c} H_{1}=Cv_{e}-\frac{1}{\gamma_{e}}({\left[{\text{Na}}^{+}\right]}_{e}+{\left[{\text{K}}^{+}\right]}_{e}-{\left[{\text{Cl}}^{-}\right]}_{e}). \end{array}$$

We fix

$$\begin{array}{c} H_{1}=-70-\frac{1}{\gamma_{e}}(10+140-5)\approx -3,258,497\mu \text{A}\cdot {\text{cm}}^{-2}. \end{array}$$

We can then express the potassium concentration in the pyramidal neuron as

$$\begin{array}{c} {\left[{\text{K}}^{+}\right]}_{e}=\gamma_{e}(v_{e}-H_{1})-{\left[{\text{Na}}^{+}\right]}_{e}+{\left[{\text{Cl}}^{-}\right]}_{e}. \end{array}$$

We proceed similarly for the GABAergic neuron: let

$$\begin{array}{c} H_{2}=Cv_{i}-\frac{1}{\gamma_{i}}({\left[{\text{Na}}^{+}\right]}_{i}+{\left[{\text{K}}^{+}\right]}_{i}), \end{array}$$

with

$$\begin{array}{c} H_{2}=-70-\frac{1}{\gamma_{i}}(10+140)\approx -2,947,024\mu \text{A}\cdot {\text{cm}}^{-2}. \end{array}$$

The potassium concentration in the GABAergic neuron is given by:

$$\begin{array}{c} {\left[{\text{K}}^{+}\right]}_{i}=\gamma_{i}(v_{i}-H_{2})-{\left[{\text{Na}}^{+}\right]}_{i}. \end{array}$$

Note that, in such a configuration, we should not model external inputs to the neurons with a constant current appearing only in the equation for the membrane potential. For example, if we add a constant external current *J* to the right hand side of Eq (1a):

$$\begin{array}{cc} & C\;\frac{dv_{e}}{dt}=-I_{\text{Na},e}-I_{K,e}-I_{\text{Cl},e}+J, \end{array}$$

Eq (2) becomes

$$\begin{array}{c} C\frac{dv_{e}}{dt}-\frac{1}{\gamma_{e}}(\frac{d{\left[{\text{Na}}^{+}\right]}_{e}}{dt}+\frac{d{\left[{\text{K}}^{+}\right]}_{e}}{dt}-\frac{d{\left[{\text{Cl}}^{-}\right]}_{e}}{dt})=J. \end{array}$$

Integrating, we see that it causes a drift of the system:

$$\begin{array}{cc} & Cv_{e}-\frac{1}{\gamma_{e}}({\left[{\text{Na}}^{+}\right]}_{e}+{\left[{\text{K}}^{+}\right]}_{e}-{\left[{\text{Cl}}^{-}\right]}_{e})=\text{constant}+Jt, \end{array}$$

which thus cannot have any steady state nor limit cycle. Instead, we modeled external inputs to the neurons using synaptic currents. We assumed a constant glutamate input on AMPA receptors, which generates sodium and potassium currents. Those currents appear both in the equation for the membrane potential and in the ones for the intracellular ion concentrations, preserving the first integrals *H*_1_ and *H*_2_. They are defined in Section (2.2.2).

#### 2.2.2 Transmembrane ion currents

The reversal potentials, which are used to compute ion currents, are typically assumed to be constant. Here, they vary with the ion concentrations, and their dependence on the corresponding ion gradient is given by the Nerst equation:

$$\begin{array}{c} E_{\text{ion}}=\frac{RT}{z_{\text{ion}}F}\text{log}(\frac{{\left[\text{ion}\right]}_{\text{extracellular}}}{{\left[\text{ion}\right]}_{\text{intracellular}}}), \end{array}$$

where *R* = 8, 314 mJ ⋅ (K ⋅ mol)^−1^ is the ideal gas constant, *T* the temperature, *F* = *N*_A_ *e* the Faraday constant and *z*_ion_ the valence.

**Pyramidal neuron**. For the pyramidal neuron, the currents generated by the sodium and potassium voltage-gated channels were modeled as in [26]:

- Fast inactivating sodium current: $I_{\text{Na},\text{FI},e}=g_{\text{Na},\text{FI},e}\;m_{e}^{3}\;h_{e}\;\left(v_{e}-E_{\text{Na},e}\right)$,
- Delayed rectifier potassium current: $I_{K,\text{DR},e}=g_{K,\text{DR},e}\;n_{e}^{4}\;\left(v_{e}-E_{K,e}\right)$.

As [26], we took into account a calcium-activated potassium current, which is involved in the afterhyperpolarization (AHP) phase of action potentials. It is defined as

$$\begin{array}{c} I_{K,\text{AHP},e}=g_{K,\text{AHP},e}\;\frac{{\left[{\text{Ca}}^{2+}\right]}_{e}}{{\left[{\text{Ca}}^{2+}\right]}_{e}+K_{\text{Ca}}}\;\left(v_{e}-E_{K,e}\right). \end{array}$$

The implementation of cotransporters was also directly taken from [26]. Those proteins perform secondary active transport: they use the favorable movement of molecules with their electrochemical gradient as an energy source to move other molecules against their gradient. The potassium-chloride transporter member 5 (KCC2) extrudes potassium and chloride (Fig 1), using the potassium gradient to maintain low intracellular chloride concentration:

$$\begin{array}{c} I_{\text{KCC}}=\frac{\rho_{\text{KCC}}}{\gamma_{e}}\;\text{log}(\frac{{\left[K^{+}\right]}_{e}{\left[{\text{Cl}}^{-}\right]}_{e}}{{\left[K^{+}\right]}_{o}{\left[{\text{Cl}}^{-}\right]}_{o}}). \end{array}$$

The Na-K-Cl cotransporter isoform NKCC1 transports sodium, potassium and chloride into the cell, with the stoichiometry 1Na:1K:2Cl (Fig 1):

$$\begin{array}{c} I_{\text{NKCC}}=\frac{1}{\gamma_{e}}\;\frac{\rho_{\text{NKCC}}}{1+\text{exp}(K_{\text{NKCC},K}-{\left[K^{+}\right]}_{o})}(\text{log}(\frac{{\left[K^{+}\right]}_{e}{\left[{\text{Cl}}^{-}\right]}_{e}}{{\left[K^{+}\right]}_{o}{\left[{\text{Cl}}^{-}\right]}_{o}})+\text{log}(\frac{{\left[{\text{Na}}^{+}\right]}_{e}{\left[{\text{Cl}}^{-}\right]}_{e}}{{\left[{\text{Na}}^{+}\right]}_{o}{\left[{\text{Cl}}^{-}\right]}_{o}})). \end{array}$$

Contrary to [26], we distinguished the sodium, potassium and chloride components of the leak current:

- Leak sodium current: *I*_Na,L,e_ = *g*_Na,L,e_(*v*_e_ − *E*_Na,e_),
- Leak potassium current: *I*_K,L,e_ = *g*_K,L,e_(*v*_e_ − *E*_K,e_),
- Leak chloride current: *I*_Cl,L,e_ = *g*_Cl,L,e_(*v*_e_ − *E*_Cl,e_).

This allowed us to measure their effect on the different ion concentrations. Similarly, we separated the sodium and potassium currents due to the excitatory autapse, assuming an equal permeability of the glutamatergic receptors to both ions:

-
$I_{\text{Na},\text{GLU},e}=\frac{g_{\text{GLU},e}}{2}s_{e}(v_{e}-E_{\text{Na},e})$ ,
-
$I_{K,\text{GLU},e}=\frac{g_{\text{GLU},e}}{2}s_{e}(v_{e}-E_{K,e})$ .

As explained in Section (2.2.1), external inputs to the pyramidal neurons were modeled with a constant glutamate input on those receptors:

-
$I_{\text{Na},D,e}=\frac{g_{D,e}}{2}\left(v_{e}-E_{\text{Na},e}\right)$ ,
-
$I_{K,D,e}=\frac{g_{D,e}}{2}\left(v_{e}-E_{K,e}\right)$ .

Those currents represent average excitatory network activity or experimental depolarizations. The inhibitory synaptic current, created by the movement of chloride ions through GABA_A_ receptors, was modeled as in [26]:

$$\begin{array}{c} I_{\text{GABA},e}=g_{\text{GABA},e}\;s_{i}\;\left(v_{e}-E_{\text{Cl},e}\right). \end{array}$$

To model the activity of the Na^+^/K^+^ ATPase, we used this expression [34, 43]:

$$\begin{array}{c} I_{\text{pump},e}=\rho_{\text{pump}}\left(v_{e}\right)(\frac{{\left[{\text{Na}}^{+}\right]}_{e}}{{\left[{\text{Na}}^{+}\right]}_{e}+K_{\text{pump},\text{Na}}}{)}^{3}(\frac{{\left[{\text{K}}^{+}\right]}_{o}}{{\left[{\text{K}}^{+}\right]}_{o}+K_{\text{pump},K}}{)}^{2}, \end{array}$$

with half-activation parameters from [35]. Based on Bouret et al. [36], we also introduced a voltage dependence of the pump maximal rate:

$$\begin{array}{c} \rho_{\text{pump}}\left(v\right)=\rho_{\text{pump},-70}\frac{f(v)}{f(-70)}, \end{array}$$

where

$$\begin{array}{c} f\left(v\right)=\frac{1+\text{tanh}(a\frac{F}{RT}v+b)}{2}. \end{array}$$

To summarize, the net currents for each ion are:

$$\begin{array}{cc} & I_{\text{Na},e}=I_{\text{Na},\text{FI},e}+I_{\text{Na},L,e}+3I_{\text{pump},e}+I_{\text{NKCC}}+I_{\text{Na},\text{GLU},e}+I_{\text{Na},D,e},\\ & I_{K,e}=I_{K,\text{DR},e}+I_{K,\text{AHP},e}+I_{K,L,e}+I_{\text{KCC}}+I_{\text{NKCC}}-2I_{\text{pump},e}+I_{K,\text{GLU},e}+I_{K,D,e},\\ & I_{\text{Cl},e}=I_{\text{Cl},L,e}-I_{\text{KCC}}-2I_{\text{NKCC}}+I_{\text{GABA},e}. \end{array}$$

**GABAergic neuron**. The sodium and potassium currents through the voltage-gated channels of the GABAergic neurons were modeled as in [33]:

- Fast inactivating Na^+^ current: $I_{\text{Na},\text{FI},i}=g_{\text{Na},\text{FI},i}\;m_{\infty ,i}^{3}\;h_{i}\left(v_{i}-E_{\text{Na},i}\right)$,
- Delayed rectifier K^+^ current: $I_{K,\text{DR},i}=g_{K,\text{DR},i}\;n_{i}^{2}\left(v_{i}-E_{K,i}\right)$.

For more details on the gating dynamics of those channels, see Section (2.2.5).

In the same way as for the pyramidal neuron, we separated the components pertaining to the different ions for the leak currents:

- Leak Na^+^ current: *I*_Na,L,i_ = *g*_Na,L,i_(*v*_i_ − *E*_Na,i_),
- Leak K^+^ current: *I*_K,L,i_ = *g*_K,L,i_(*v*_i_ − *E*_K,i_),

for the glutamatergic synaptic currents:

-
$I_{\text{Na},\text{GLU},i}=\frac{g_{\text{GLU},i}}{2}s_{e}(v_{i}-E_{\text{Na},i})$ ,
-
$I_{K,\text{GLU},i}=\frac{g_{\text{GLU},i}}{2}s_{e}(v_{i}-E_{K,i})$ ,

and for the glutamatergic synaptic currents which model an average external input to the GABAergic neuron:

-
$I_{\text{Na},D,i}=\frac{g_{D,i}}{2}\left(v_{i}-E_{\text{Na},i}\right)$ ,
-
$I_{K,D,i}=\frac{g_{D,i}}{2}\left(v_{i}-E_{K,i}\right)$ .

The Na^+^/K^+^ ATPase current is given by the same sigmoidal function as for the pyramidal neuron:

$$\begin{array}{c} I_{\text{pump},i}=\rho_{\text{pump}}\left(v_{i}\right)(\frac{{\left[{\text{Na}}^{+}\right]}_{i}}{{\left[{\text{Na}}^{+}\right]}_{i}+K_{\text{pump},\text{Na}}}{)}^{3}(\frac{{\left[{\text{K}}^{+}\right]}_{o}}{{\left[{\text{K}}^{+}\right]}_{o}+K_{\text{pump},K}}{)}^{2}. \end{array}$$

We obtained the following sodium and potassium net currents:

$$\begin{array}{cc} & I_{\text{Na},i}=I_{\text{Na},\text{FI},i}+I_{\text{Na},P,i}+I_{\text{Na},L,i}+3I_{\text{pump},i}+I_{\text{Na},\text{GLU},i}+I_{\text{Na},D,i},\\ & I_{K,i}=I_{K,\text{DR},i}+I_{K,L,i}-2I_{\text{pump},i}+I_{K,\text{GLU},i}+I_{K,D,i}. \end{array}$$

#### 2.2.3 Calcium concentration in the pyramidal neuron

The conductance of the calcium-activated potassium current *I*_K,AHP,e_ is determined by the pyramidal neuron’s intracellular calcium concentration. As in [26], we modeled the dynamics of this concentration with Eq (1h) from Wang [41]:

$$\begin{array}{cc} \frac{d{\left[{\text{Ca}}^{2+}\right]}_{e}}{dt}= & -\frac{\gamma_{e}}{2}\;I_{\text{Ca},e}-\frac{{\left[{\text{Ca}}^{2+}\right]}_{e}}{\tau_{\text{Ca}}}. \end{array}$$

*I*_Ca,e_ represents high threshold calcium current and is given by:

$$\begin{array}{c} I_{\text{Ca},e}=\;g_{\text{Ca},e}\;m_{\infty ,\text{Ca}}\;(v_{e}-E_{\text{Ca},e}). \end{array}$$

It is a transmembrane current, but for simplicity we did not take into account its effect on the pyramidal neuron’s membrane potential. Unlike in [26], we used the same conversion factor *γ*_e_ as for the other ions, for consistency. The second term models various extrusion and buffering mechanism, with a first order decay process.

#### 2.2.4 Diffusion of extracellular potassium

As in [26], the following term appears in the equation for the extracellular potassium Eq (1p):

$$\begin{array}{c} I_{K,\text{diff}}=\varepsilon ({\left[{\text{K}}^{+}\right]}_{o}-K_{\text{bath}}). \end{array}$$

It accounts for passive diffusion of extracellular potassium, but also, in a simplistic way, for the buffering of this ion by glial cells. This motivated the use of a large value of the diffusion coefficient *ϵ* (see Table 2), which is a conservative choice.

#### 2.2.5 Gating variables dynamics

Gating variables represent the state of activation of voltage-gated channels. In Section 2.2.2 and Section 2.2.3, they scale the maximal conductance of those channels.

**Pyramidal neuron**. For the pyramidal neuron, the dynamics of the potassium and sodium gating variables is given by Eqs (1b)–(1d). As in [26], we used a simplified version of the Traub-Miles model [44] due to Wei et al. [28]:

$$\begin{array}{cc} & \alpha_{m,e}=0.32\frac{v_{e}+54}{1-\text{exp}(-\frac{v_{e}+54}{4})},\\ & \beta_{m,e}=0.28\frac{v_{e}+27}{\text{exp}(\frac{v_{e}+27}{5})-1},\\ & \alpha_{h,e}=0.128\;\text{exp}\left(-\frac{v_{e}+50}{18}\right),\\ & \beta_{h,e}=\frac{4}{1+\text{exp}(-\frac{v_{e}+27}{5})},\\ & \alpha_{n,e}=0.032\frac{v_{e}+52}{1-\text{exp}(-\frac{v_{e}+52}{5})},\\ & \beta_{n,e}=0.5\;\text{exp}\left(-\frac{v_{e}+57}{40}\right). \end{array}$$

Eq (1h) describes the dynamics of the intracellular calcium concentration, needed to compute the conductance of the calcium-activated potassium channels. As in [26], we assumed that the state of the calcium channels depends instantaneously on the voltage, according to this expression by Wang [41] with parameter values from Gutkin et al. [40]:

$$\begin{array}{c} m_{\infty ,\text{Ca}}=\frac{1}{1+\text{exp}(-\frac{v_{e}+25}{2.5})}. \end{array}$$

**GABAergic neuron**. For the GABAergic neuron, we replaced the Wang-Buzsáki model [32] with a more recent one by Golomb et al. [33], which is specific to fast-spiking cortical interneurons:

$$\begin{array}{cc} & m_{\infty ,i}=\frac{1}{1+\text{exp}(-\frac{v_{i}-\left(-24\right)}{11.5})},\\ & h_{\infty ,i}=\frac{1}{1+\text{exp}(-\frac{v_{i}-\left(-58.3\right)}{6.7})},\\ & \tau_{h,i}=0.5+\frac{14}{1+\text{exp}(-\frac{v_{i}-\left(-60\right)}{-12})},\\ & n_{\infty ,i}=\frac{1}{1+\text{exp}(-\frac{v_{i}-\left(-12.4\right)}{6.8})},\\ & \tau_{n,i}=(0.087+\frac{11.4}{1+\text{exp}(\frac{v_{i}+14.6}{8.6})})(0.087+\frac{11.4}{1+\text{exp}(-\frac{v_{i}-1.3}{18.7})}). \end{array}$$

The sodium activating variable is assumed to be at its steady state value *m*_∞,i_. The dynamics of the sodium inactivating variable *h*_i_ and of the potassium activating variable *n*_i_ is given in Eqs (1k) and (1l).

#### 2.2.6 Mutations of Na_V_1.1

Na_V_1.1 is mainly expressed in GABAergic neurons and Na_V_1.1 mutations affect mainly these neurons [2]. Thus, we assumed in our simulations that the pyramidal neuron is unaffected by mutations of this channel. As we shall see, to model FHM-3 or epilepsy we only modified two parameters: the maximal conductance *g*_Na,FI,i_ of the fast-inactivating sodium current and the maximal conductance *g*_Na,P,i_ of the persistent sodium current, which we introduce in the case of migraine.

**FHM-3 mutations**. Increased persistent sodium current is a common effect of most Na_V_1.1 FHM-3 mutations [6–9]. To model them, we partially replaced the GABAergic neuron’s fast inactivating sodium current *I*_Na,FI,i_ with a persistent sodium current *I*_Na,P,i_. We kept the sum of their maximal conductances constant: *g*_Na,FI,i_ + *g*_Na,P,i_ = 112.5 mS · cm^−2^. We modeled persistent current with the following expression:

$$\begin{array}{c} I_{\text{Na},P,i}=g_{\text{Na},P,i}m_{\infty ,i}^{3}\left(v_{i}+8\right)\left(v_{i}-E_{\text{Na},i}\right). \end{array}$$

Note that it does not include the inactivation mechanism represented by the variable *h*_i_ for the fast inactivating current. We also shifted the voltage dependence of its activation to more negative potentials [45, 46]. Let *p*_Na,P_ be the percentage of maximal voltage-gated sodium conductance corresponding to persistent current:

$$\begin{array}{c} p_{\text{Na},P}=\frac{g_{\text{Na},P,i}}{g_{\text{Na},\text{FI},i}+g_{\text{Na},P,i}}\cdot 100. \end{array}$$

To model Na_V_1.1 FHM-3 mutations, we tested values up to *p*_Na,P_ = 20%; see Section (3.1).

**Epileptogenic mutations**. Epileptogenic mutations of Na_V_1.1 cause a loss of function of the channel [1, 2]. To model them, we decreased the maximal conductance of the GABAergic neuron’s fast inactivating sodium current *g*_Na,FI,i_. Based on what was experimentally observed in *Scn1a*^+/-^ mice [1], we set it to 40% of its default value, reflecting the condition of heterozygosis and the expression of other voltage-gated sodium channels than Na_V_1.1 in GABAergic neurons:

$$\begin{array}{c} g_{\text{Na},\text{FI},i}=0.4\cdot 112.5=\left[45\right]\text{mS}\cdot {\text{cm}}^{-2}. \end{array}$$

### 2.3 Mouse lines, preparation of brain slices and electrophysiological recordings

Whole-cell patch clamp recordings were performed with the F1 generation of crosses between heterozygous *Scn1a*^+/-^ knock-out mice (C57BL/6J-CD1 85:15%) [1, 47] and C57BL/6J GAD67-GFPΔneo knock-in mice (which label GABAergic neurons with GFP [48]), comparing P15-18 double heterozygous (*Scn1a*^+/-^_GAD67-GFPΔneo) and GAD67-GFPΔneo littermates. Optogenetic experiments for CSD induction and juxtacellular recordings were performed with P25-30 hemizygous transgenic VGAT-hChR2(H134R)/tdtomato mice (VGAT-ChR2 in the text) in the C57BL/6J background, in which channelrhodopsin-H134R/tdtomato is specifically expressed in GABAergic neurons. They were the F1 generation obtained by crossing hemizygous females loxP-STOP-loxP-hChR2(H134R)-tdtomato (Ai27D, B6.Cg-Gt(ROSA)26Sortm27.1(CAG-COP4*H134R/tdTomato)Hze/J; Jackson lab, JAX, n°012567) [49] with hemizygous Viaat-Cre transgenic males, in which selective Cre recombinase expression in GABAergic neurons is driven by the vesicular GABA transporter (VGAT) promoter (B6.FVB-Tg(Slc32a1-Cre)2.1Hzo/FrkJ; JAX n°017535), line 2.1 in [50]). Offspring was genotyped by PCR, following the standard JAX protocols for VGAT-ChR2 mice and using our standard protocol for *Scn1a*^+/-^ mice [29, 47], or selecting fluorescent pups monitored with a Dual Fluorescent Protein flashlight (NightSea).

Brain slices of the somatosensory cortex were prepared as previously described [25, 29, 47, 51]. Briefly, mice were killed by decapitation under isofluorane anesthesia, the brain was quickly removed and placed in ice-cold artificial cerebrospinal fluid (ACSF), which contained (mM): NaCl 129, MgSO_4_ 1.8, KCl 3, CaCl_2_ 1.6, NaHCO_3_ 21, NaH_2_PO_4_ 1.25 and glucose 10 bubbled with 95% O_2_ 5% CO_2_. Coronal slices (380 μm thick) were prepared with a vibratome (Microm HM650V or Leica VT1200S) in ice-cold ACSF, placed in a holding chamber at room temperature in ACSF continuously bubbled with 95% O_2_ 5% CO_2_, and used after one hour of recovery period. One slice at the time was placed in the recordings chamber (Warner Instruments, USA) and neurons were visualized by epifluorescence and infrared video microscopy with a Nikon Eclipse FN1 equipped with epifluorescence DIC optics and a CCD camera. Spatial optogenetic stimulation for activating ChR2 and inducing neuronal firing in a specific area was obtained illuminating brain slices through a 4x objective with 473 nm blue light generated using a white light source (130W Intensilight, Nikon) connected with a light guide containing a 420 nm UV blocker filter (series 2000, Lumatec, Germany) to a digital micromirror device (DMD)-based patterned photostimulator (Polygon 400, Mightex), whose output was filtered with a 475/50 filter (Semrock) and the light delivered to the objective with a FF685-Di02 dichroic beamsplitter (Semrock) [29].

Patch-clamp recordings were performed with a Multiclamp 700B amplifier, Digidata 1440a digitizer and pClamp 10.2 software (Axon Instruments, USA); signals were filtered at 10 kHz and acquired at 50 kHz. Whole-cell recordings of neuronal firing were done at 28°C in current-clamp mode applying the bridge balance compensation; the external recording solution was ACSF (see above) and the internal solution contained (mM): K-gluconate, 120; KCl, 15; MgCl_2_, 2; EGTA, 0.2; Hepes, 10; Na_2_ATP, 2; Na_2_GTP 0.2; leupeptine, 0.1; P-creatine 20, pH 7.25 with KOH. Patch pipettes were pulled from borosilicate glass capillaries; they had resistance of 2.5-3.0 MΩ and access resistance of 5-10 MΩ. We held the resting potential at −70 mV by injecting the appropriate holding current, and neuronal firing was induced injecting depolarizing current pulses of increasing amplitude. Neurons with unstable resting potential and/or unstable firing were discarded from the analysis. Juxtacellular-loose patch recordings of neuronal firing were performed in voltage-clamp mode perfusing slices with modified mACSF at 34°C (which contained (in mM): 125 NaCl, 3.5 KCl, 1 CaCl_2_, 0.5 MgCl_2_, 1.25 NaH_2_PO_4_, 25 NaHCO_3_ and 25 glucose, bubbled with 95% O_2_—5% CO_2_) and using the same pipettes used for whole cell experiments, but filled with ACSF. Recordings were performed from GABAergic neurons of Layer 2-3, identified by their fluorescence and morphology. Fast-spiking neurons were selected for the analysis of whole cell recordings, identified by their firing properties (short, < 1 ms, action potentials with pronounced after-hyperpolarization, non-adapting discharges reaching several hundred Hz of maximal firing frequency).

For the statistical analysis, the reported *n* is the number of cells recorded; each experiment was performed using at least 3 animals. Statistical tests were performed with Origin (Origin Lab. Corp, USA), using the two-tailed non parametric Mann-Whitney U test. Differences were considered significant at *p* < 0.05.

The original raw data is available at https://doi.org/10.5281/zenodo.4926119.

## 3 Results

### 3.1 Na_V_1.1 FHM-3 mutations can lead to CSD initiation via extracellular potassium build-up also when neuronal input-output features are not modified

#### 3.1.1 Persistent sodium current (*I*_Na,P,i_) in GABAergic neurons amplifies spiking-induced modifications of extracellular ion concentrations, even without modifications of their firing frequency

We first focused on Na_V_1.1 FHM-3 mutations, modeled by the increase of *I*_Na,P,i_ for the GABAergic neuron which is a common effect of most of those mutations [6–9], and on their effect on the features of the GABAergic neuron itself. To investigate the latter point, we obtained simulations with a version of the model in which the influence of the pyramidal neuron on the GABAergic neuron, through synaptic connections or through variations of extracellular ion concentrations, was removed.

**Fig 2:**
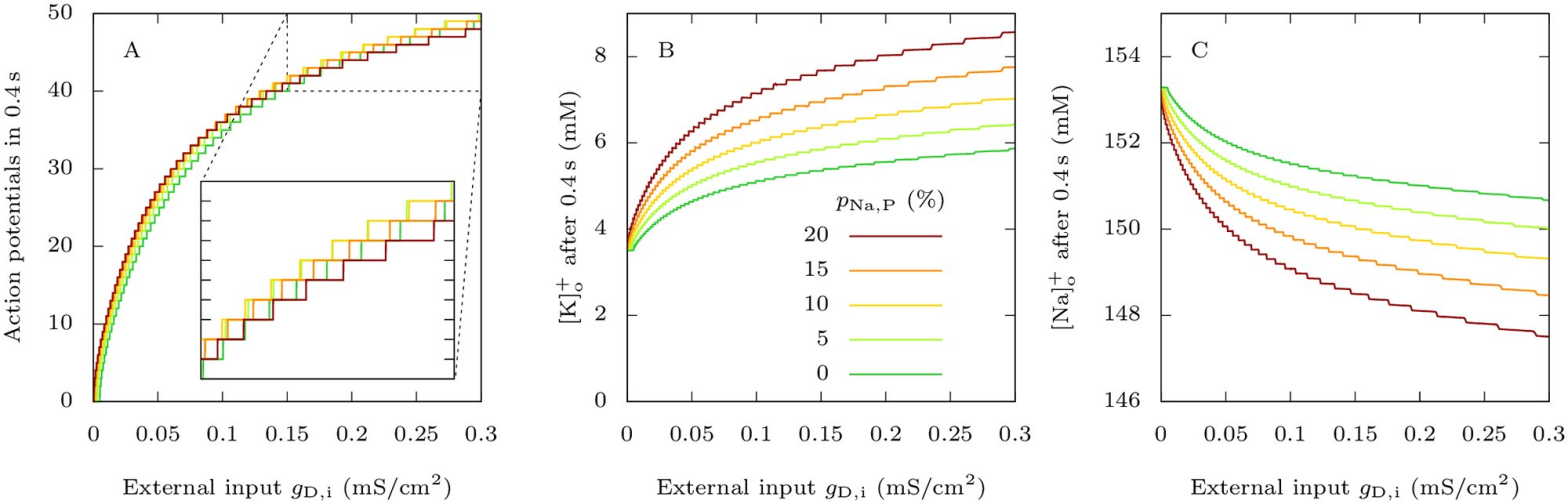
Effect of FHM-3 mutations on the firing properties of the GABAergic neuron. For different values of *p*_Na,P_ (defined in Section 2.2.6), which we increase to model FHM-3 mutations, we applied to the GABAergic neuron 0.4 s long excitatory external inputs of conductances *g*_D,i_. For each *p*_Na,P_ value, we took as initial condition the steady state in the absence of external input, i.e. when *g*_D,i_ = 0 mS · cm^−2^. **A**: Number of action potentials, considered as overshooting spikes (i.e. exceeding 0 mV of peak membrane potential). **B**: Extracellular potassium concentration at the end of the 0.4 s long simulations. **C**: Extracellular sodium concentration at the end of the 0.4 s long simulations.

To study how *I*_Na,P,i_ modifies the excitability of the GABAergic neuron, we computed the following input-output relationships: number of action potentials elicited during the application of a depolarizing external current of fixed duration versus the conductance of this current (Fig 2A). We found that an increase of *I*_Na,P,i_ reduces the rheobase (i.e. minimal external input necessary to trigger at least one action potential): 0.0004 mS ⋅ cm^−2^ when *p*_Na,P_ = 20% (migraine condition) instead of 0.0051 mS ⋅ cm^−2^ when *p*_Na,P_ = 0% (control condition), where *p*_Na,P_ is defined in Section 2.2.6. This is consistent with a decrease of the rheobase observed experimentally in neocortical mouse neurons transfected with hNa_V_1.1-L1649Q, a pathogenic mutant of the Na_V_1.1 channel associated with FHM [7]. However, in contrast to [7], in our model FHM-3 mutations do not substantially increase the GABAergic neuron’s firing frequency. With external inputs *g*_D,i_ up to approximately 0.1 mS ⋅ cm^−2^, an increase of *p*_Na,P_ consistently leads to a moderate increase of the number of action potentials generated, but this is not the case for stronger external inputs. Interestingly, there may be variability among GABAergic neurons, concerning how their firing frequency is affected by FHM-3 mutations. In a novel FHM-3 knock-in mouse model, Freilinger et al. (personal communication; see acknowledgments) observed an increase of frequency in fast-spiking GABAergic neurons, but no significant difference in regular spiking interneurons, although they both express Na_V_1.1.

On the other hand, persistent sodium current clearly enhances the accumulation of extracellular potassium (Fig 2B) and the uptake of extracellular sodium (Fig 2C) due to the firing of the GABAergic neuron, even when the number of action potentials is similar or slightly smaller than in the control condition. For example, for an external input of conductance *g*_D,i_ = 0.3 mS · cm^−2^ and *p*_Na,P_ = 20%, with 48 spikes in 0.4 s we obtained an extracellular potassium concentration of 8.6 mM (145% increase) and an extracellular sodium concentration of 147.5 mM (3.7% decrease). However, when *p*_Na,P_ = 0%, there were 49 spikes in 0.4 s but only 5.9 mM of extracellular potassium (68% increase) and still 150.7 mM of extracellular sodium (1.7% decrease) at the end of the simulation.

**Fig 3:**
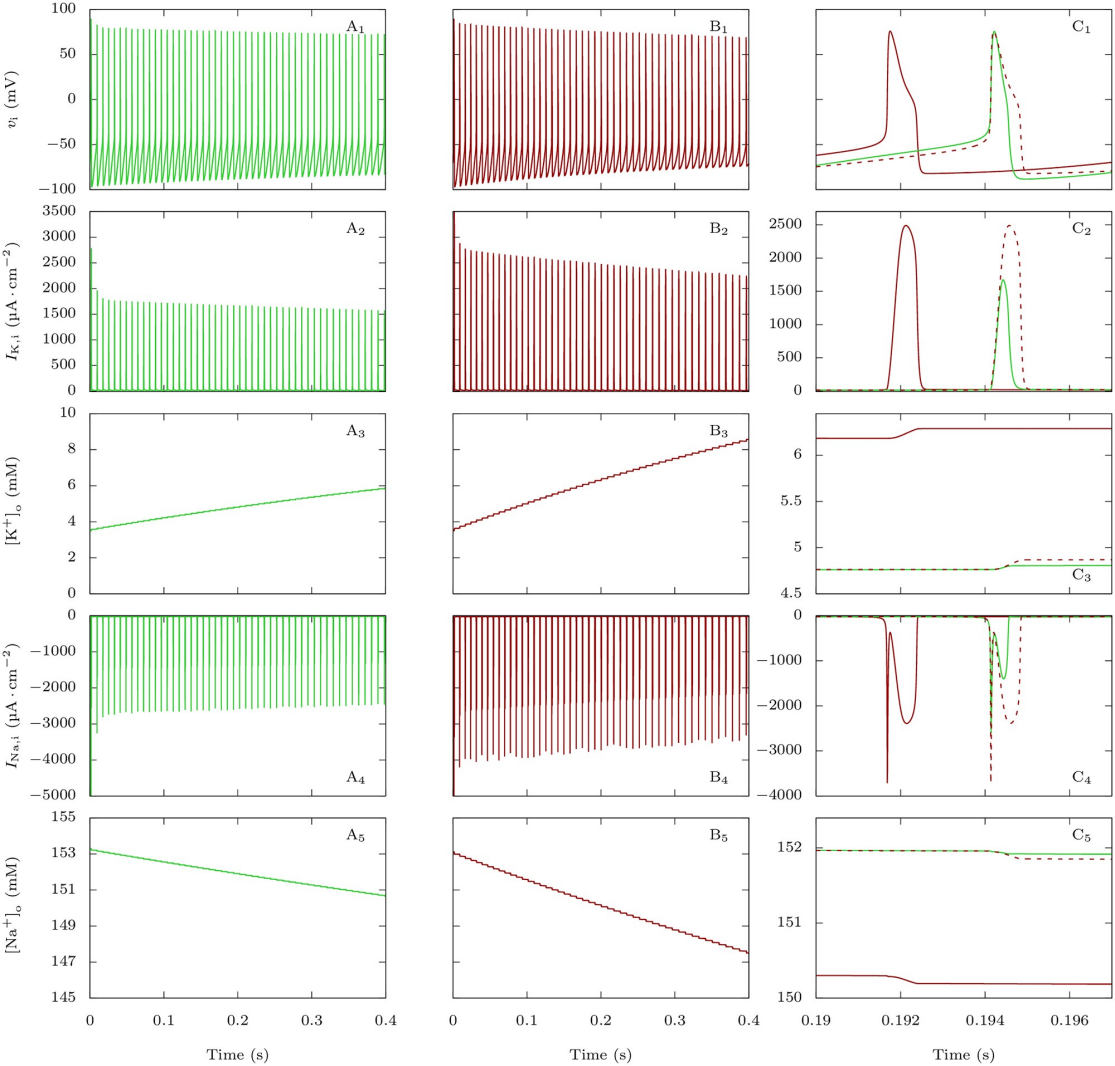
Effect of FHM-3 mutations on the firing and action currents of the GABAergic neuron, and correlation with the dynamics of [K^+^]_o_ and [Na^+^]_o_. Plots of the voltage (action potentials; upper row), total potassium current (second row), extracellular potassium concentration (third row), total sodium current (fourth row) and extracellular sodium concentration (bottom row) corresponding to simulations displayed in Fig 2, for the condition in which *g*_D,i_ = 0.3 mS · cm^−2^. **A**: No persistent sodium current: *p*_Na,P_ = 0%. **B**: *p*_Na,P_ = 20%. **C**: Zoom showing the 25^th^ action potential of A (in green) and B (in red). The dashed lines are the curves obtained with *p*_Na,P_ = 20% shifted to allow a better comparison with the curves obtained with *p*_Na,P_ = 0%.

This is a counterintuitive result that we have better investigated comparing detailed features of action potentials and underlying ionic currents for those parameter values: *g*_D,i_ = 0.3 mS · cm^−2^ and *p*_Na,P_ = 0% or 20% (Fig 3). With increased *I*_Na,P,i_, simulations showed larger action potential half width (0.365 ms for the 25^th^ action potential with *p*_Na,P_ = 0% and 0.565 ms with *p*_Na,P_ = 20%, Fig 3C_1_). However, modifications of action potential features were relatively small compared to the increase of *I*_Na,P,i_. We reasoned that the *I*_Na,P,i_ increase could induce a larger activation of potassium channels during the action potentials, able to limit the modifications of action potential features, but leading to larger potassium currents and consequent spiking-induced extracellular potassium build up. Indeed, the plots of the potassium action currents show a large increase when *I*_Na,P,i_ is higher (0.520 μA ⋅ cm^−2^ for the 25^th^ action potential in the discharge with *p*_Na,P_ = 0%, 1.311 μA ⋅ cm^−2^ with *p*_Na,P_ = 20%, Fig 3C_2_), leading to a larger accumulation of extracellular potassium at each action potential (Fig 3A_3_, 3B_3_ and 3C_3_). As expected, action sodium currents show a large increase as well, in particular during the repolarization phase (0.478 μA ⋅ cm^−2^ for the 25^th^ action potential in the discharge with *p*_Na,P_ = 0%, 1.284 μA ⋅ cm^−2^ with *p*_Na,P_ = 20%, Fig 3C_4_), leading to a consequent larger decrease of extracellular sodium concentration at each action potential (Fig 3A_5_, 3B_5_ and 3C_5_).

#### 3.1.2 Dynamics of neuronal firing at CSD initiation

In this section, we show how FHM-3 mutations in the GABAergic neuron influence the entry of the pyramidal neuron into depolarization block, which we consider as the initiation of a CSD, when the two neurons are coupled. We depolarized them with the same external inputs *g*_D,e_ = *g*_D,i_ = 0.3 mS · cm^−2^, and we compared the control condition (*p*_Na,P_ = 0%) with a pathological one (*p*_Na,P_ = 15%).

**Fig 4:**
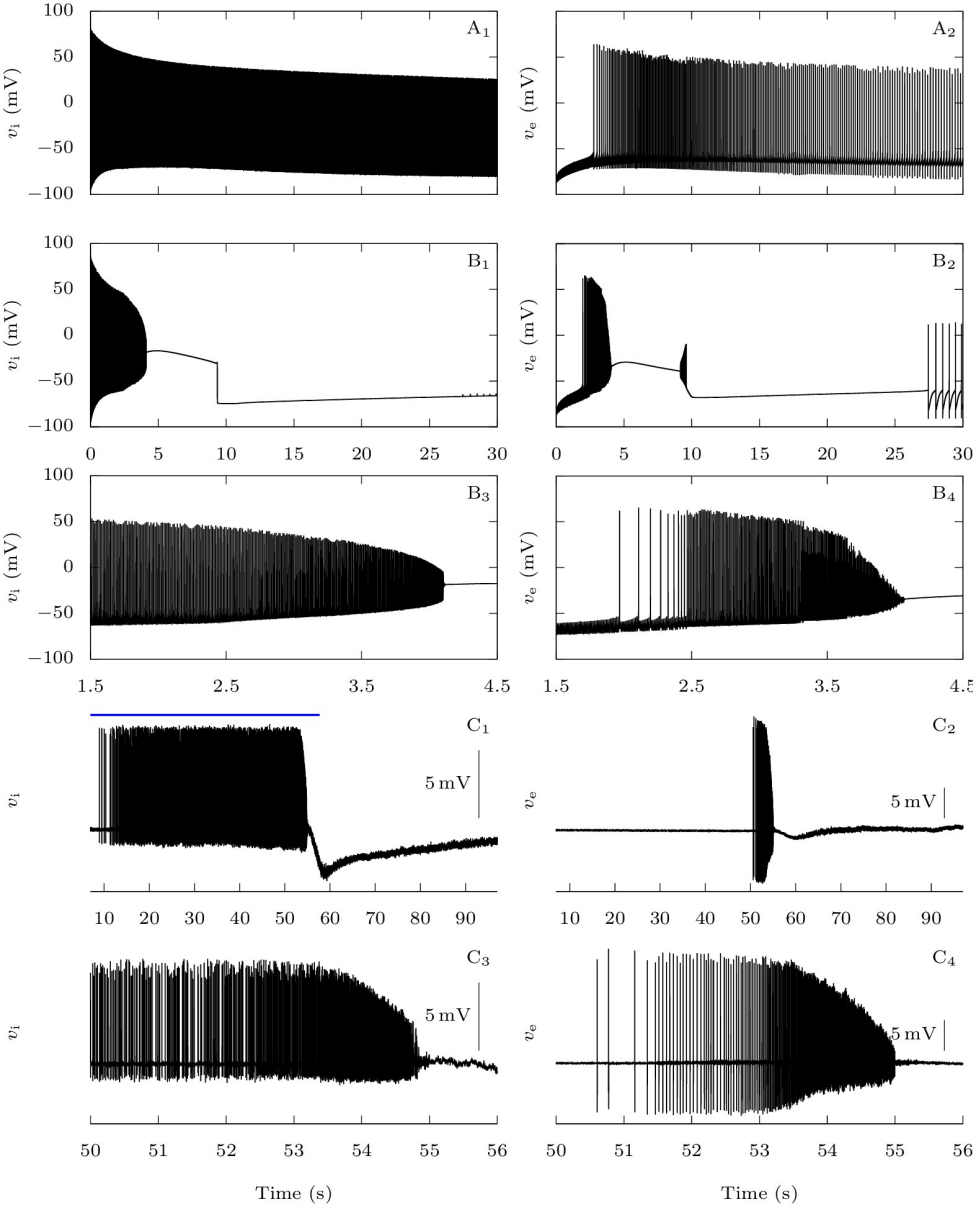
Effect of the FHM-3 mutation on CSD initiation: Representative time traces. **A-B**: Model simulations. Starting from neurons at rest, similarly to Fig 2, we stimulated them with constant excitatory conductances *g*_D,i_ = *g*_D,e_ = 0.3 mS · cm^−2^ for 30 s. **A**: No persistent sodium current for the GABAergic neuron (*p*_Na,P_ = 0%). **B**: Pathological condition (*p*_Na,P_ = 15%). **C**: Experimental data. Representative dual juxtacellular-loose patch voltage recordings of a GABAergic interneuron (**C_1_ and C_3_**) and a pyramidal neuron (**C_2_ and C_4_**) showing the dynamics of the firing at the site of CSD initiation; CSD was induced by spatial optogenetic activation of GABAergic neurons and is the slow negative deflection observable in C_1_ and C_2_. The blue bar in C_1_ shows the optogenetic stimulation with blue light.

**Fig 5:**
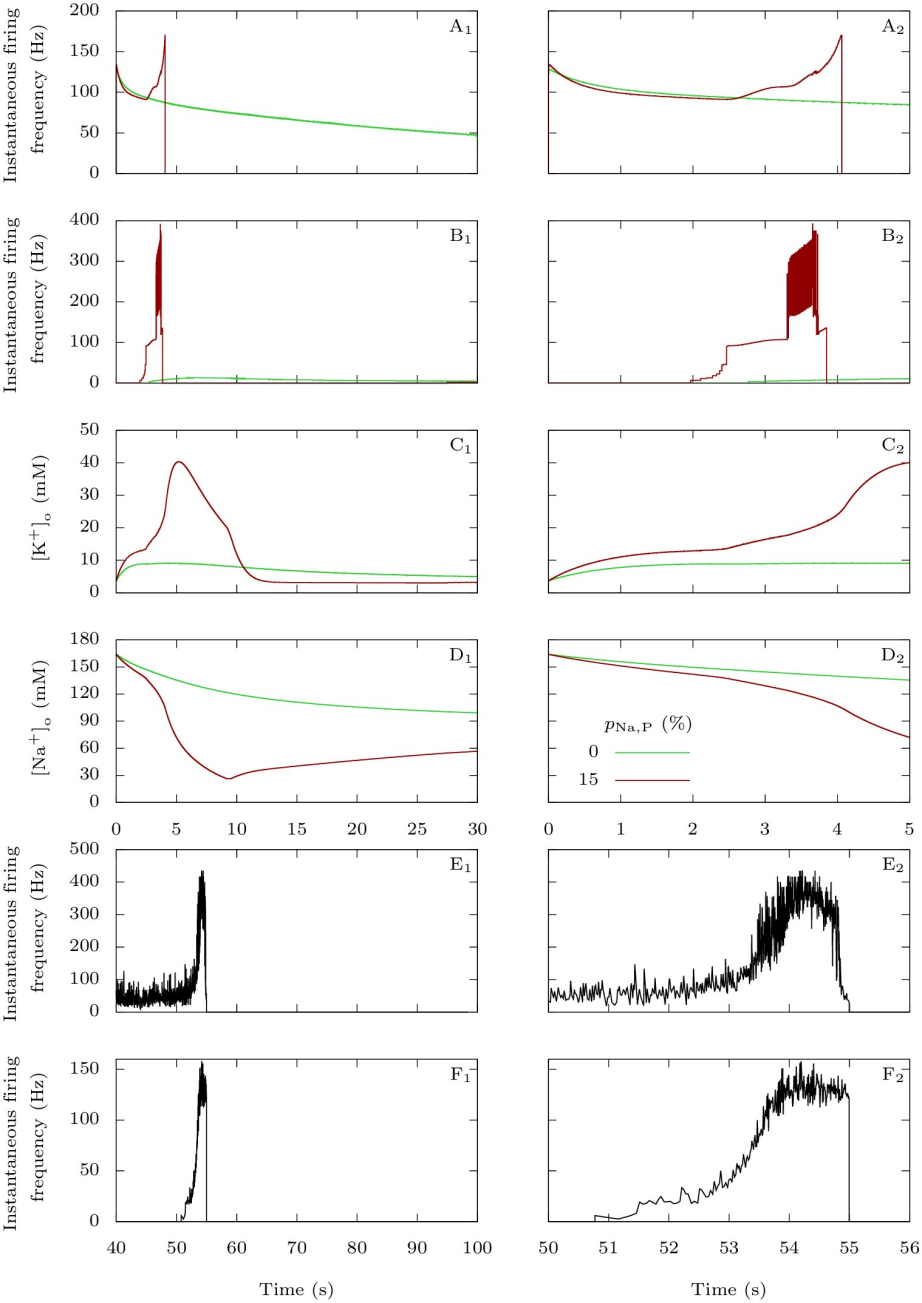
Behavior of the firing frequency and of the extracellular ion concentrations before the onset of CSD. **A-D**: Model simulations. For the first 5 s of the simulations presented in Fig 4, we compared the pathological situation (*p*_Na,P_ = 15%) with the physiological one (*p*_Na,P_ = 0%). **A**: Instantaneous firing frequency, i.e. inverse of the interspike interval, for the GABAergic neuron. **B**: Instantaneous firing frequency for the pyramidal neuron. **C**: Extracellular potassium concentration. **D**: Extracellular sodium concentration. **E-F**: Experimental data. **E**: Instantaneous firing frequency of the loose patch recording of the GABAergic neuron shown in Fig 4C_1_ and 4C_3_. **F**: Instantaneous firing frequency of the loose patch recording of the pyramidal neuron shown in Fig 4C_2_ and 4C_4_. CSD was induced by the optogenetic stimulation of the GABAergic neuron; the two phases in the firing dynamics, with lower and higher frequency respectively, are evident and similar to those observed in the simulation. Although the first phase was longer for GABAergic neurons (55.3 ± 4.5 s, *n* = 7) than for pyramidal neurons (1.6 ± 0.2 s, *n* = 5; *p* = 0.006 Mann-Whitney test), the second phase had similar duration (2.0 ± 0.2 s for GABAergic neurons; 1.7 ± 0.3 s for pyramidal neurons; *p* = 0.28, Mann-Whitney test) and firing frequency was not significantly different (mean instantaneous firing frequency in the first phase was 48.4 ± 6.8 Hz for GABAergic neurons and 32.4 ± 8.5 Hz for pyramidal neurons, *p* = 0.19 Mann-Whitney test; in the second phase it was 349 ± 36 Hz for GABAergic neurons and 214 ± 57 Hz for pyramidal neurons, *p* = 0.06 Mann-Whitney test).

In the first case, tonic spiking of the GABAergic neuron begins immediately while there is a latency of a few seconds before the pyramidal neuron also starts to generate repetitive action potentials (Fig 4A). Those initial subthreshold oscillations demonstrate the inhibitory nature of the GABAergic neuron in a physiological situation. As expected, the firing frequency is larger in the GABAergic neuron than in the pyramidal one (Fig 5A and 5B). With pathological persistent sodium current in the GABAergic neuron, the voltage traces are very different (Fig 4B). As in the control condition, there is a delay before sustained firing of the pyramidal neuron, but both the GABAergic interneuron and the pyramidal neuron show two phases of activity, and during the first phase extracellular potassium rises faster and to a larger extent than in control (Fig 5C). This is coherent with the results obtained on the GABAergic neuron alone in Section 3.1.1. Shortly after the pyramidal neuron begins to spike, we observe an increase of its firing frequency to towards an initial plateau, together with a steeper slope for the increase in extracellular potassium concentration. This is followed by a further increase of the pyramidal neuron’s firing frequency. The second phase leads to the beginning of depolarization block for both neurons, while extracellular potassium continues to grow. Notably, sodium overload during the depolarization block (Fig 5D) can contribute to silencing of firing.

In order to study experimentally the dynamics of the firing of both GABAergic interneurons and pyramidal neurons at the site of CSD initiation, we performed pairs of juxtacellular-loose patch voltage recordings, inducing CSD by spatial optogenetic activation of GABAergic neurons as in [29]. Although this method of induction does not completely reproduce the condition of the simulation, it mimics hyperexcitability of the GABAergic neurons and allows recordings at the pre-determined site of CSD initiation, which cannot be performed with other experimental models of CSD. Notably, the firing dynamics was similar to that obtained with the simulation. We found that GABAergic neurons began to fire at the beginning of the illumination, whereas pyramidal neurons began to fire later during the illumination, just for few seconds before CSD initiation (Figs 4C, 5E and 5F). As in the simulation, the firing dynamics of both GABAergic and pyramidal neurons showed two phases, a first phase with lower firing frequency (which was on average 34.6-fold longer for the GABAergic neurons) and a second phase of similar duration (few seconds) for the two types of neurons, in which firing frequency increased on average 8.3±1.8-fold for GABAergic neurons and 8.1±2.5-fold for pyramidal neurons, leading to depolarization block. Although few GABAergic neurons fired at very high frequency, instantaneous frequency was on average not different between GABAergic and pyramidal neurons.

#### 3.1.3 Persistent sodium current in GABAergic neurons reduces the threshold for CSD initiation

**Fig 6:**
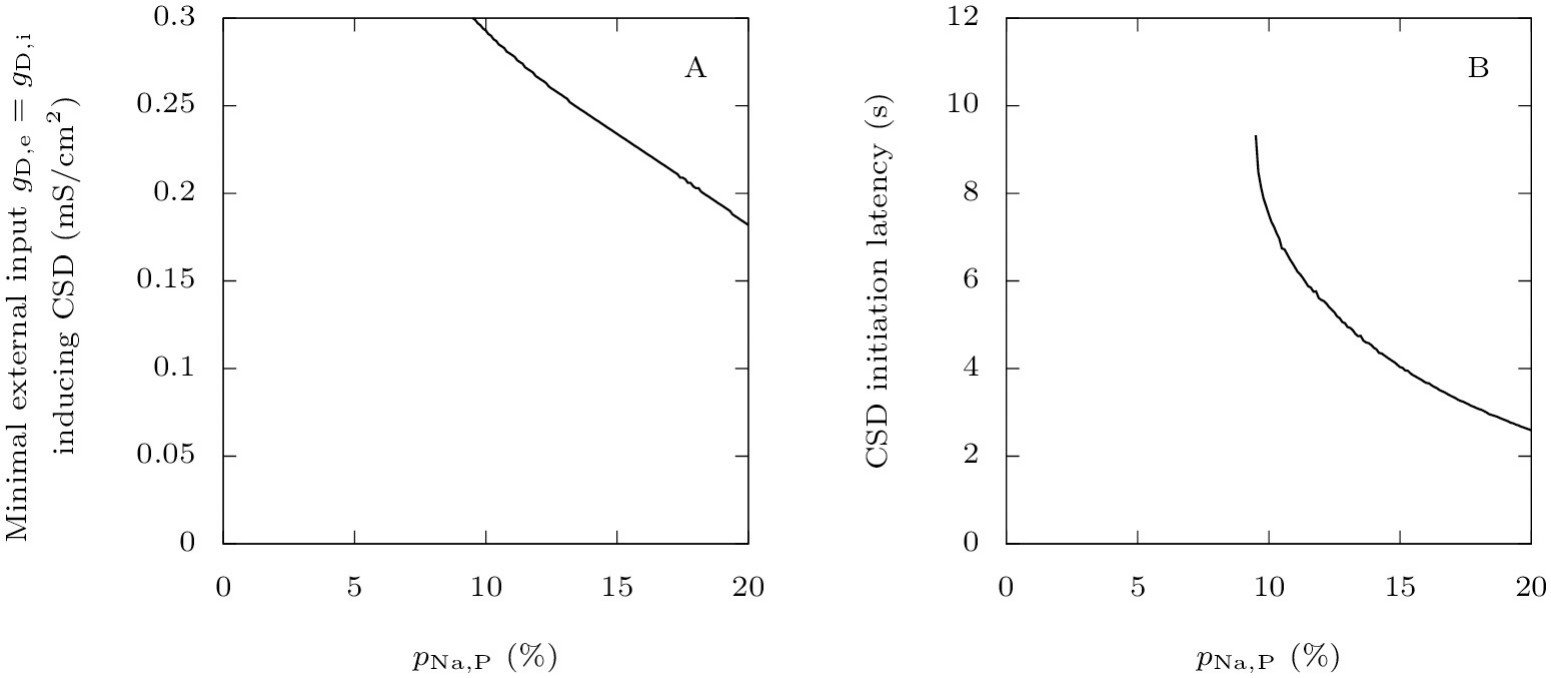
Effect of FHM-3 mutations on CSD initiation. **A**: We show here how increasing the GABAergic neuron’s persistent sodium current influences the minimal external input necessary to induce a depolarization block in the pyramidal neuron, varying *p*_Na,P_ from 0 to 20%. We used as external inputs constant excitatory conductances, equal for both neurons, taking values up to *g*_D,i_ = *g*_D,e_ = 0.3 mS · cm^−2^. We considered that beyond these values, inputs would be unrealistically strong, leading to a response of the model that would not be interpretable. Again, we chose as initial condition the steady state when the neurons are not stimulated. We defined a depolarization block by the absence of oscillations of amplitude larger than 5 mV of the voltage for at least half a second, with the additional condition that it must be between −55 mV and −20 mV at the end of this interval. For each *p*_Na,P_, we estimated the smaller external input for which this criterion is met for the pyramidal neuron with a bisection method. **B**: Starting from neurons at rest, for given large external inputs *g*_D,e_ = *g*_D,i_ = 0.3 mS · cm^−2^, we computed the time it takes for a CSD to be initiated, if it is initiated at all.

More generally, we studied whether those findings are robust to modifications of the value of parameter *p*_Na,P_ in the pathological case. This is important since we do not know precisely which level of persistent sodium current best models the effects of FHM-3 mutations. Migraine is an episodic disorder, which means that patients exhibit symptoms only during attacks: a trigger factor causes the shift from a physiological state to a pathological state [12]. In our model, FHM-3 mutations reduce the threshold for this transition. Indeed, the more persistent sodium current in the GABAergic neuron, the smaller the minimal external input necessary to initiate CSD (Fig 6A). This is consistent with our recent experimental work where it was shown that bath application of Hm1a, a toxin which mimics the effects of FHM-3 mutations by increasing persistent sodium current, can lead to spontaneous CSD initiation in mouse brain slices [29]. Moreover, we found that, for a given large external input, persistent sodium current decreases the latency to CSD (Fig 6B). Here also, our simulations agree qualitatively with experimental data. Indeed, optogenetic activation of GABAergic neurons induces CSD earlier in slices perfused with Hm1a than in control slices [29]. Those two points are also supported by experiments on the knock-in mouse model of the L1649Q FHM-3 mutation: the threshold for eliciting CSD by electrical stimulation was substantially lowered in heterozygous mice, and the latency from KCL application to the onset of CSD was significantly reduced (Freilinger et al., personal communication; see acknowledgments).

Very close to the threshold that is approximated in Fig 6A, the response oscillates between non-CSD and CSD solutions, when gradually increasing the external input. This shows great sensitivity of the model at the transition between physiological and pathological behaviors. We cannot assert whether this is a numerical effect or a property inherent to the model. In any case, such a phenomenon is not surprising given the dimensionality of the model and its multiple time scales. Similar issues already arise in much simpler conductance-based models, e.g. FitzHugh-Nagumo, Morris-Lecar or a 2D reduction of Hodgkin-Huxley, which are type-2 neuron models for which the firing threshold may not be well defined [52]. The approximation of the threshold for CSD initiation that we have obtained is nevertheless sufficient to study the roles of persistent sodium current and extracellular ion concentrations. Computing the exact threshold, if it exists, is challenging, and we do not consider it relevant for the purpose of the present study. This is an interesting question for future work, where we will focus on reducing our current model while keeping its most salient features.

#### 3.1.4 The accumulation of extracellular potassium is crucial for CSD initiation

**Fig 7:**
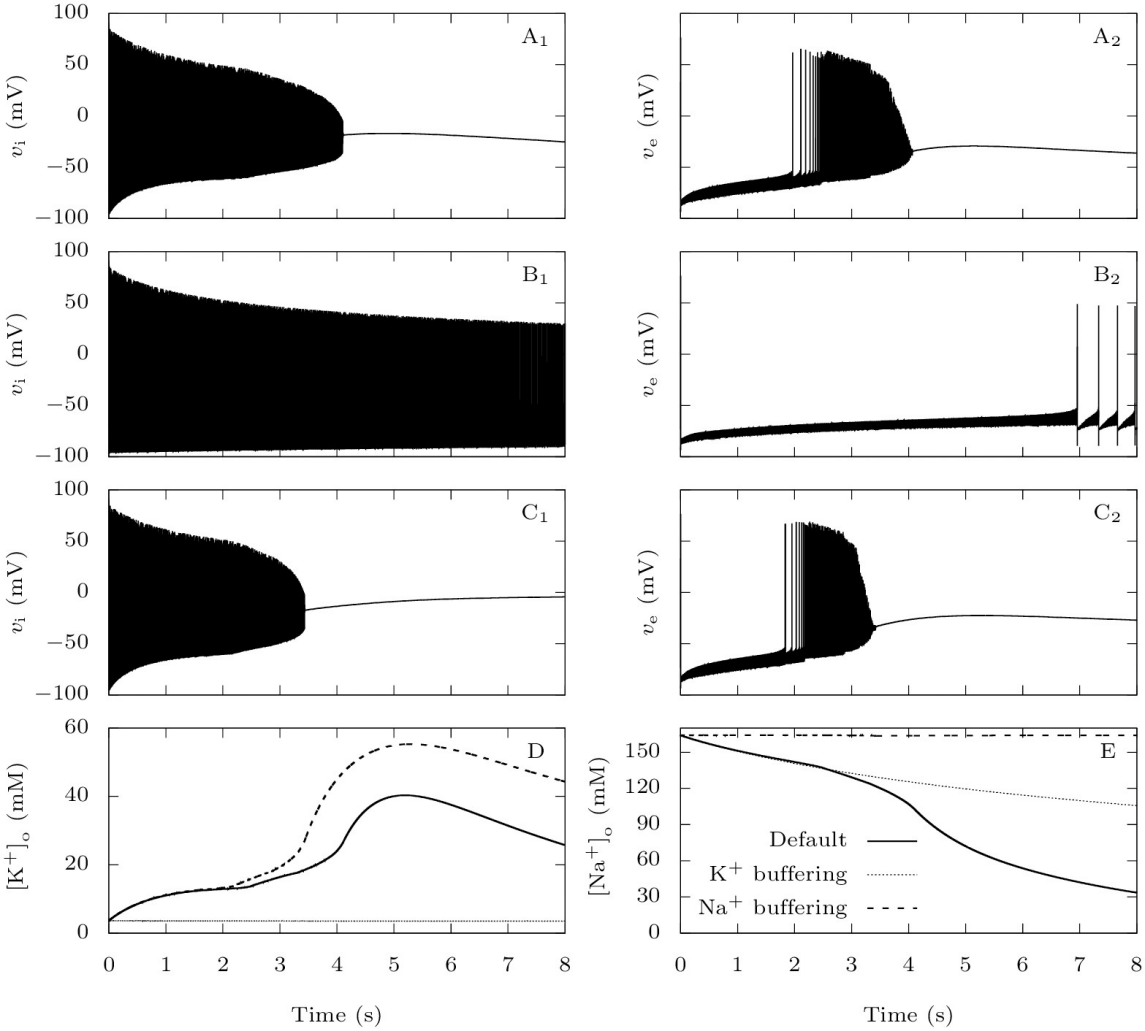
The roles of extracellular ions in CSD initiation: Representative time traces. When Na_V_1.1 carries a FHM-3 mutation, modeled by *p*_Na,P_ = 15%, we tested the effect of keeping either extracellular potassium or sodium constant using unrealistically high diffusion rates. We depolarized the neurons at rest with constant excitatory conductances *g*_D,i_ = *g*_D,e_ = 0.3 mS · cm^−2^ for 30 s. **A**: Voltage traces for the default parameters. **B**: Voltage traces when *ε* = 0.1 ms^−1^ to simulate a strong buffering of extracellular potassium. **C**: Voltage traces when there is a strong buffering of extracellular sodium. To implement it, we introduced the diffusion term *I*_diff,Na_ = *ε*_Na_([Na^+^]_o_ − Na_bath_) in the equation for extracellular sodium, with *ε*_Na_ = 0.1 ms^−1^ and Na_bath_ equal to the steady-state extracellular sodium in the absence of external input. **D**: [K^+^]_o_ traces in the cases described in A-C. **E**: [Na^+^]_o_ traces in the cases described in A-C.

**Fig 8:**
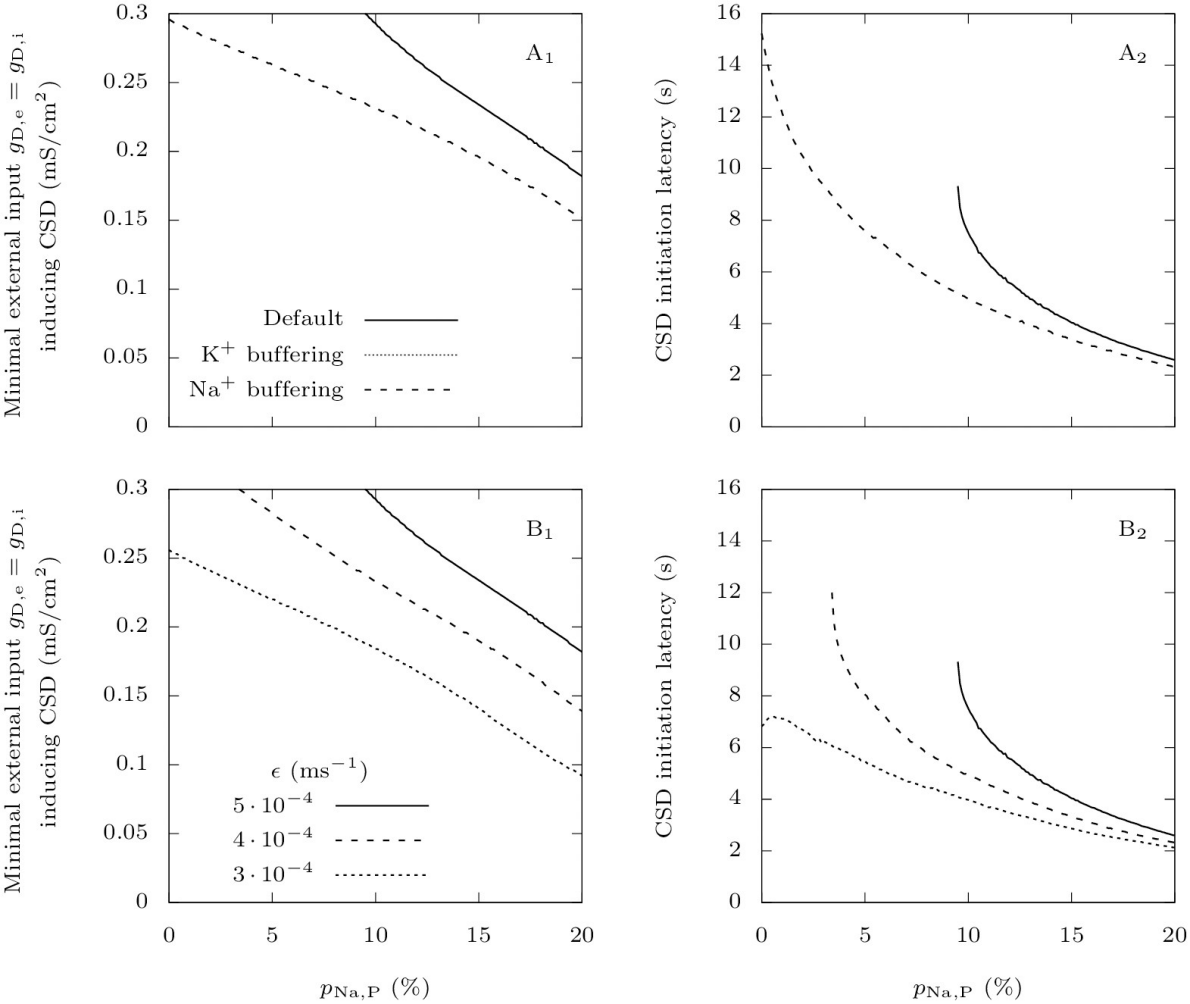
The roles of extracellular ions in CSD initiation. Similarly to Fig 6, we show in the left panel the minimal external input necessary to induce a depolarization block in the pyramidal neuron, and in the right panel the latency to CSD. **A**: We tested the effect of keeping either extracellular potassium or sodium approximately constant using the same method as for Fig 7. **B**: We tested the effect of reducing the extracellular potassium diffusion rate. The default value of this parameter is *ε* = 5 ⋅ 10^−4^ ms^−1^.

In our model, the two neurons are coupled through synaptic connections and through variations in extracellular ion concentrations. The synaptic connection from the GABAergic neuron to the pyramidal one is inhibitory in all the tested conditions (see Section 3.3). Modifications of extracellular ion concentrations can thus be the cause of the large increase of firing frequency that precedes the depolarization block observed in the pathological case (Fig 5B). In the first seconds of the simulation, both extracellular potassium and sodium concentrations are substantially modified (Fig 5C and 5D), but the relative change is much more important for potassium, consistent with the simulations with the isolated GABAergic neuron (Fig 2). A rise in extracellular potassium increases the reversal potential for this ion, which is a depolarizing effect, while a decay of the extracellular sodium has the opposite effect. This suggests that potassium plays a major role in promoting CSD initiation. To confirm it, we implemented unrealistically strong buffering of either extracellular potassium or sodium, to keep their concentration constant (Fig 7A and 7B). In the same pathological setting as in Fig 5B, we applied strong external inputs *g*_D,i_ = *g*_D,e_ = 0.3 mS · cm^−2^ to the neurons. We observe that only the strong buffering of extracellular potassium prevents CSD initiation (Fig 7B). More generally, similarly to (Fig 6), we computed the thresholds for CSD initiation and the latencies to CSD for strong external inputs (Fig 8A). Remarkably, keeping the extracellular potassium concentration low completely prevents CSD initiation over the examined ranges of persistent current and external inputs. This is not the case with sodium; in fact the strong buffering of its extracellular concentration even facilitates CSD initiation, and leads to a larger increase of extracellular potassium concentration.

We also tested smaller values of the extracellular potassium diffusion rate. This can model, in a simplistic way, the contribution of other GABAergic neurons to the accumulation of extracellular potassium, or a less efficient buffering by the glial network. As expected, it reduces the threshold for CSD initiation and CSD is ignited earlier for a given stimulus (Fig 8B).

### 3.2 Na_V_1.1 epileptogenic mutations strongly decrease the inhibitory role of GABAergic neurons

#### 3.2.1 Reduced sodium current in GABAergic neurons makes them more susceptible to depolarization block

As with the FHM-3 mutations (Section 3.1.1), we first investigated the effects of epileptogenic mutations on the GABAergic neuron itself, when it does not interact with the pyramidal neuron. To model epileptogenic mutations of Na_V_1.1, we reduced the GABAergic neuron’s fast-inactivating sodium maximal conductance, as explained in Section 2.2.6.

**Fig 9:**
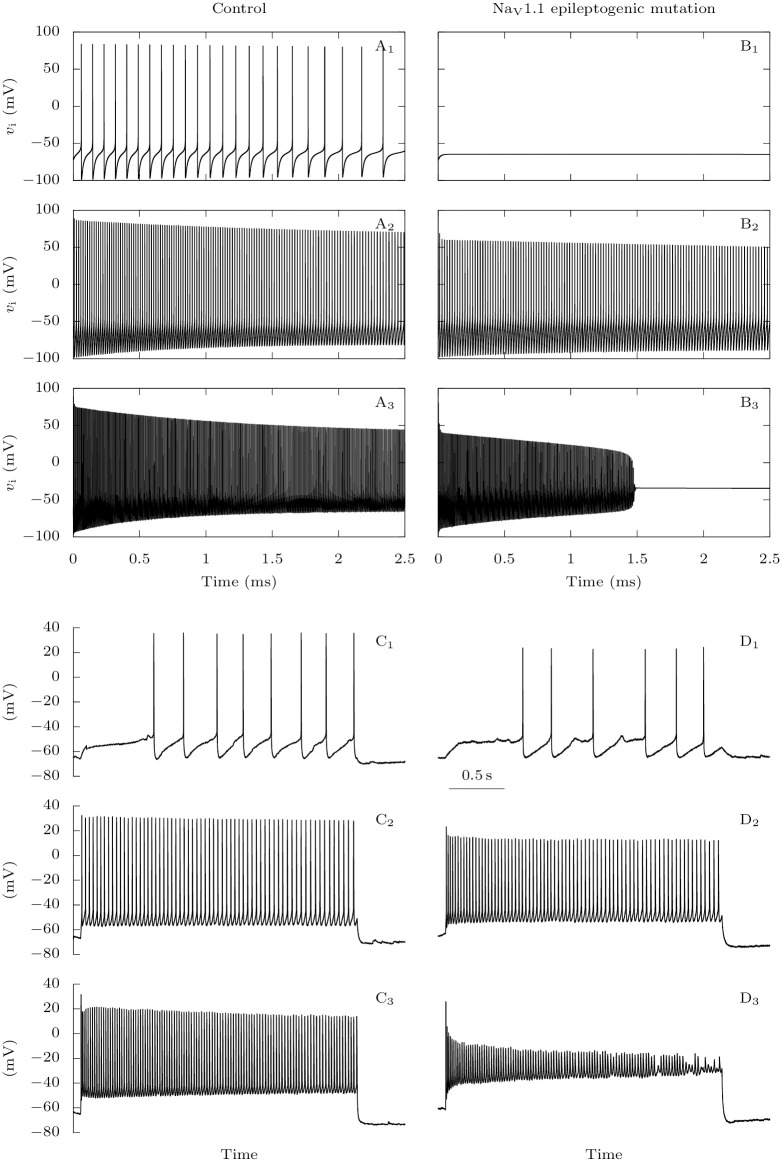
Effect of Na_V_1.1 epileptogenic mutations on the firing of GABAergic neurons: Representative traces. **A-B**: Model simulations. We depolarized the GABAergic neuron at rest with constant excitatory conductances *g*_D,i_ = 0.0075 (**A_1_**, **B_1_**), *g*_D,i_ = 0.05 (**A_2_**, **B_2_**) or *g*_D,i_ = 0.5 mS · cm^−2^ (**A_3_**, **B_3_**), for the default parameters (**A**: control condition), or when the sodium fast-inactivating maximal conductance is reduced to 40% of its default value (**B**: Na_V_1.1 epileptogenic mutation). **C-D**: Experimental data. Action potential discharges elicited in a fast spiking GABAergic layer 2-3 cortical neuron from a wild type mouse (**C**) or a *Scn1a*^+/-^ mouse (**D**), injecting depolarizing current steps of 10 pA (**C_1_,D_1_**), 100 pA (**C_2_,D_2_**) or 260 pA (**C_3_,D_3_**).

**Fig 10:**
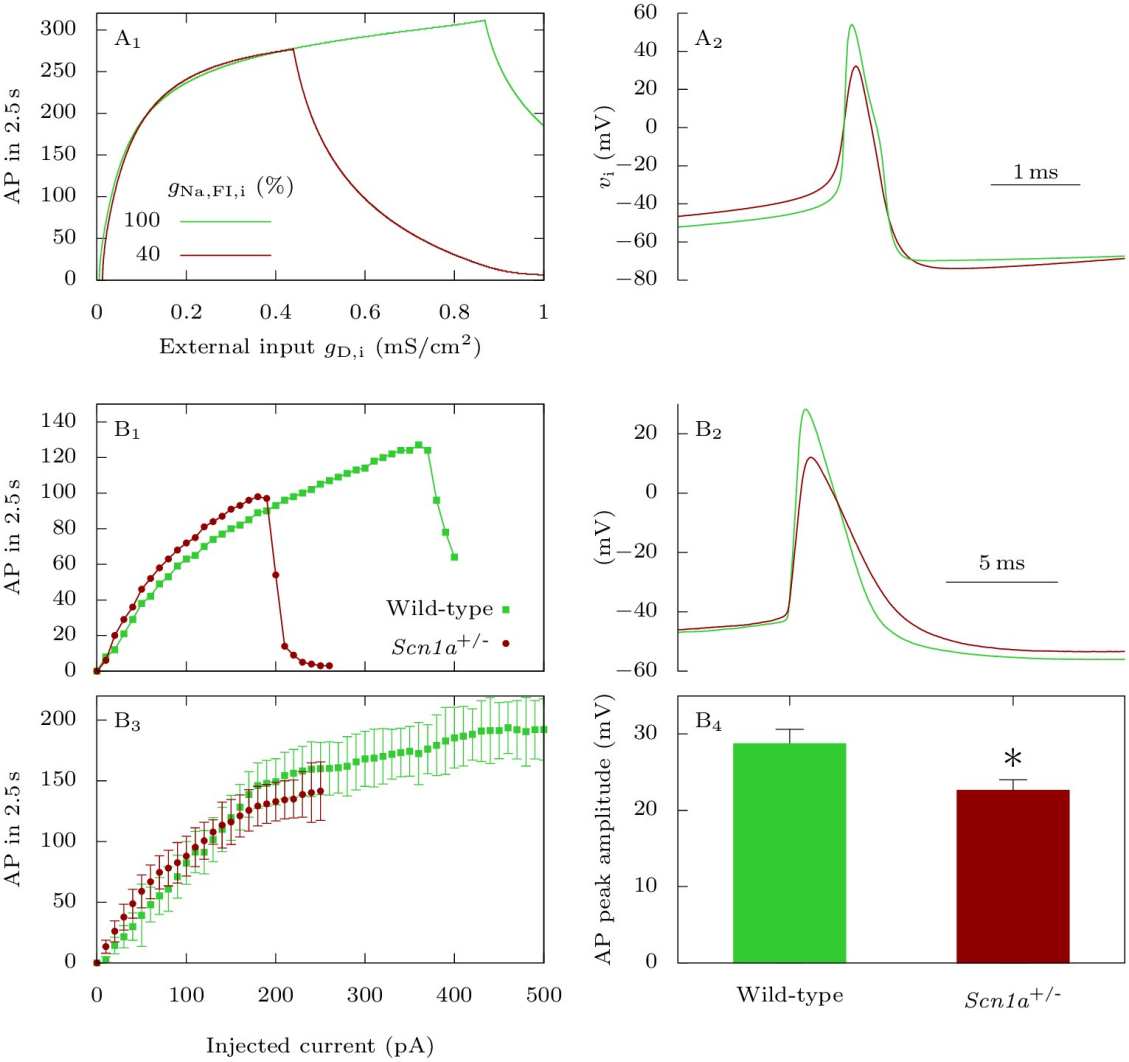
Effect of Na_V_1.1 epileptogenic mutations on the firing of GABAergic neurons: Input-output relationship and action potential amplitude. **A**: Model simulations. We applied to the GABAergic neuron at rest 2.5 s long excitatory external inputs of conductances *g*_D,i_, for the default parameters (wild-type), or for a reduced sodium fast-inactivating maximal conductance *g*_Na,FI,i_ (epileptogenic mutations). **A_1_**: Number of action potentials. **A_2_**: Representative action potentials, for *g*_D,i_ = 0.3 mS · cm^−2^. **B**: Experimental data. **B_1_**: Representative fast spiking neurons recorded in cortical slices from *Scn1a*^+/-^ mice and wild type littermates, selected for having similar input-output curves with lower injected current, showing that the *Scn1a*^+/-^ neuron was more prone to depolarization block (sharp decrease of action potential number). **B_2_**: Comparison of the first suprathreshold action potential recorded in the neurons selected for panel B1. **B_3_**: Mean input-output relationships for fast spiking neurons recorded from *Scn1a*^+/-^ (*n* = 7) and wild type littermates (*n* = 7), the number of elicited action potentials was not different, but depolarization block was induced with lower depolarizing current (the traces in which there was depolarization block were excluded, to avoid the influence of depolarization block in the evaluation of the number of action potentials elicited with larger depolarizing currents). **B_4_**: Mean peak amplitude (absolute value) of the first suprathreshold action potential for fast spiking neurons recorded from *Scn1a*^+/-^ (22.6 ± 1.5 mV, *n* = 7) and wild type littermates (28.7 ± 2.0 mV, *n* = 7); *p* = 0.044 Mann-Whitney test. * *p* < 0.05.

The simulations show that in this condition the rheobase is slightly increased, action potential amplitude is decreased and depolarization block is induced by smaller depolarizing external inputs (Figs 9A, 9B and 10A). Notably, action potential firing frequency shows just very small modifications in the input-output relationship (Fig 10A_1_), in which a slight increase is surprisingly observed for the epileptic condition between *g*_D,i_ = 0.2 and *g*_D,i_ = 0.4 mS · cm^−2^. We observed similar modifications in experimental traces, comparing cortical layer 2-3 fast spiking GABAergic neurons recorded in heterozygous *Scn1a* knock-out mice (*Scn1a*^+/-^), model of the developmental and epileptic encephalopathy Dravet syndrome [1, 2], and wild type littermates as control (Figs 9C, 9D and 10B). Notably, the comparison of single representative neurons with similar input-output relationships showed that, although firing frequency could be slightly larger for the *Scn1a*^+/-^ neuron, depolarization block was induced by a smaller depolarizing current (Figs 9C, 9D and Fig 10B_1_) and action potential amplitude was reduced (Fig 10B_2_). The comparison of average features did not disclose modifications of firing frequency before the induction of depolarization block in *Scn1a*^+/-^ (Fig 10B_3_), but depolarization block was consistently induced with smaller depolarizing currents. Of note, for obtaining these average input-output curves, we excluded for each cell the traces in which there was depolarization block, to avoid the influence of depolarization block in the evaluation of firing frequency with larger depolarizing currents. To statistically compare action potential amplitude, we quantified the mean amplitude of the first suprathreshold action potential, which was reduced in *Scn1a*^+/-^ mice (Fig 10B_4_).

**Fig 11:**
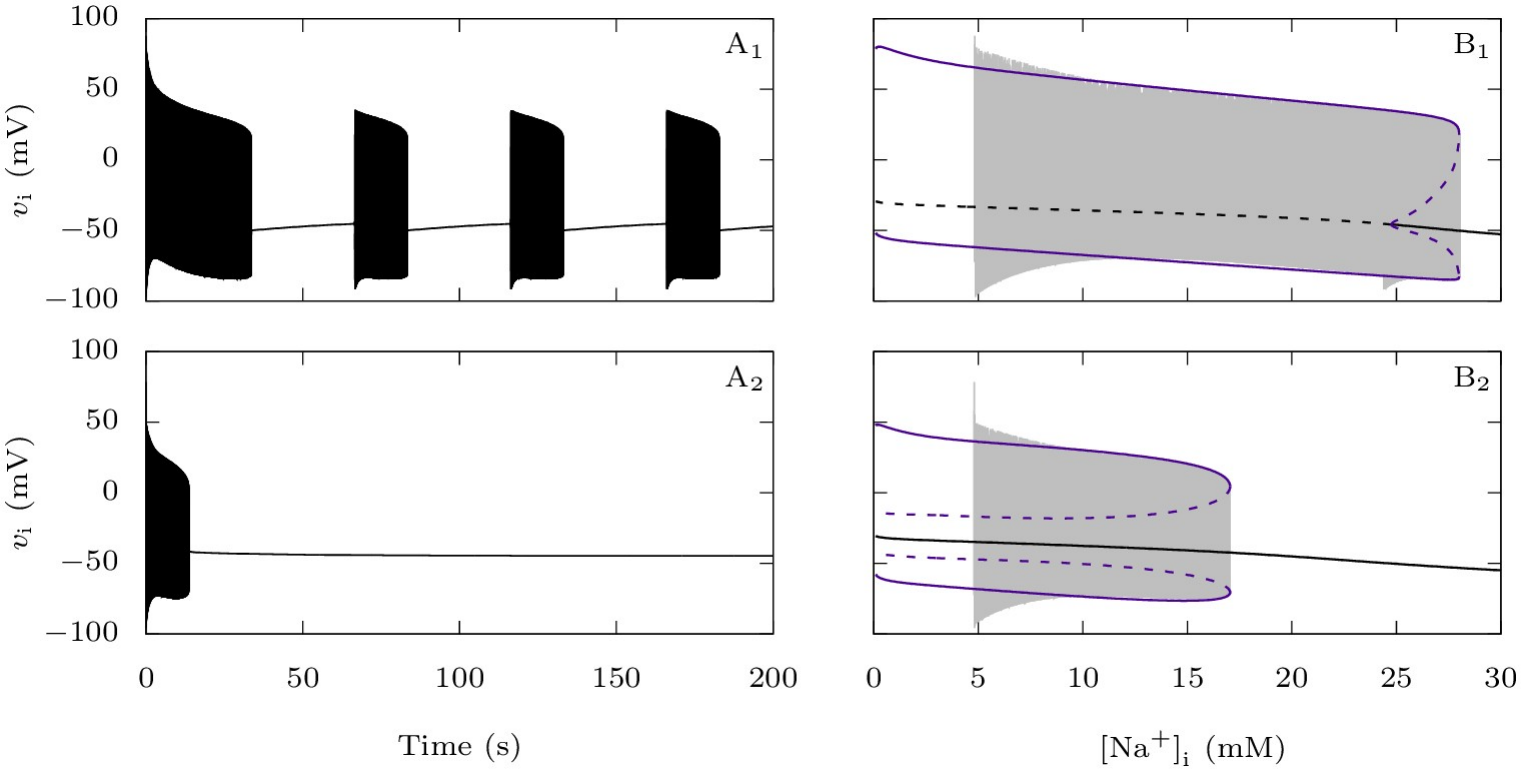
Effect of Na_V_1.1 epileptogenic mutations on the GABAergic neuron bifurcation structure. We considered the intracellular sodium concentration as a slow variable and we studied the corresponding fast subsystem where this concentration is a parameter. We focused here on the case where *g*_D,i_ = 0.3 mS · cm^−2^. **A**: Voltage trace of the full system starting from a neuron at rest, for the default parameter values (**A_1_**) or when the fast-inactivating sodium maximal conductance is reduced to 40% of its default value (**A_2_**). **B**: Trajectory of A_1_ (**B_1_**) or A_2_ (**B_2_**), projected onto the ([Na^+^]_i_, *v*_i_) plane and bifurcation diagram of the fast subsystem with respect to the intracellular sodium concentration. We represented steady-states with black lines and limit cycles with purple lines.

To better understand the enhanced transition from the firing regime to depolarization block of the GABAergic neuron when Na_V_1.1 carries an epileptogenic loss of function mutation, which we observed in Fig 10A_1_, we studied it as a *dynamic bifurcation* phenomenon [53]. Even though system (1) is not explicitly slow-fast, it is clear from its time traces that it has different time scales. We considered the sodium concentration in the GABAergic neuron [Na^+^]_i_ as a slow variable, and analyzed the corresponding *fast subsystem* where [Na^+^]_i_ is a parameter, with or without epileptogenic mutation. In Fig 11A, we show the voltage traces of the complete system when applying an excitatory conductance *g*_D,i_ = 0.3 mS · cm^−2^ to the GABAergic neurons at rest. In Fig 11B, we projected the same solutions onto the ([Na^+^]_i_, *v*_i_) plane, superimposed onto the bifurcation diagram of the fast subsystem with respect to [Na^+^]_i_, what is called a *slow-fast dissection* [54]. In the wild-type case, the solution first follows limit cycles of large amplitude, which corresponds to repetitive firing, while the sodium slowly increases (Fig 11B_1_). For [Na^+^]_i_ approximately 28 mM there is a fold of limit cycle bifurcation, which causes the solution to jump to a stable steady state. This is the beginning of a quiescent phase, where the neuron does not spike while [Na^+^]_i_ decreases slowly until a Hopf bifurcation is encountered, where the steady state becomes unstable. This causes the solution to return to the large-amplitude limit cycles. In this way, the solution alternates between active and quiescent phases (Fig 11A_1_), a neuronal behavior typically referred to as *bursting*. It is not surprising since bursting is a possible behavior of the model we have used to describe the gating dynamics of the GABAergic neuron’s voltage-gated channels [33]. The subHopf/fold-cycle hysteresis loop which enables this bursting is not preserved for the reduced value of fast-inactivating sodium maximal conductance which we use to model Na_V_1.1’s epileptogenic mutations (Fig 11B_2_). In this case, the solution of the complete system also first follows large-amplitude limit cycles while [Na^+^]_i_ slowly increases. However, once it reaches the fold of limit cycle bifurcation and jumps to the stable steady state, it does not leave it anymore: the GABAergic neuron completely stops producing action potentials (Fig 11A_2_).

#### 3.2.2 Pyramidal neurons firing frequency suddenly increases when GABAergic neurons enter depolarization block

**Fig 12:**
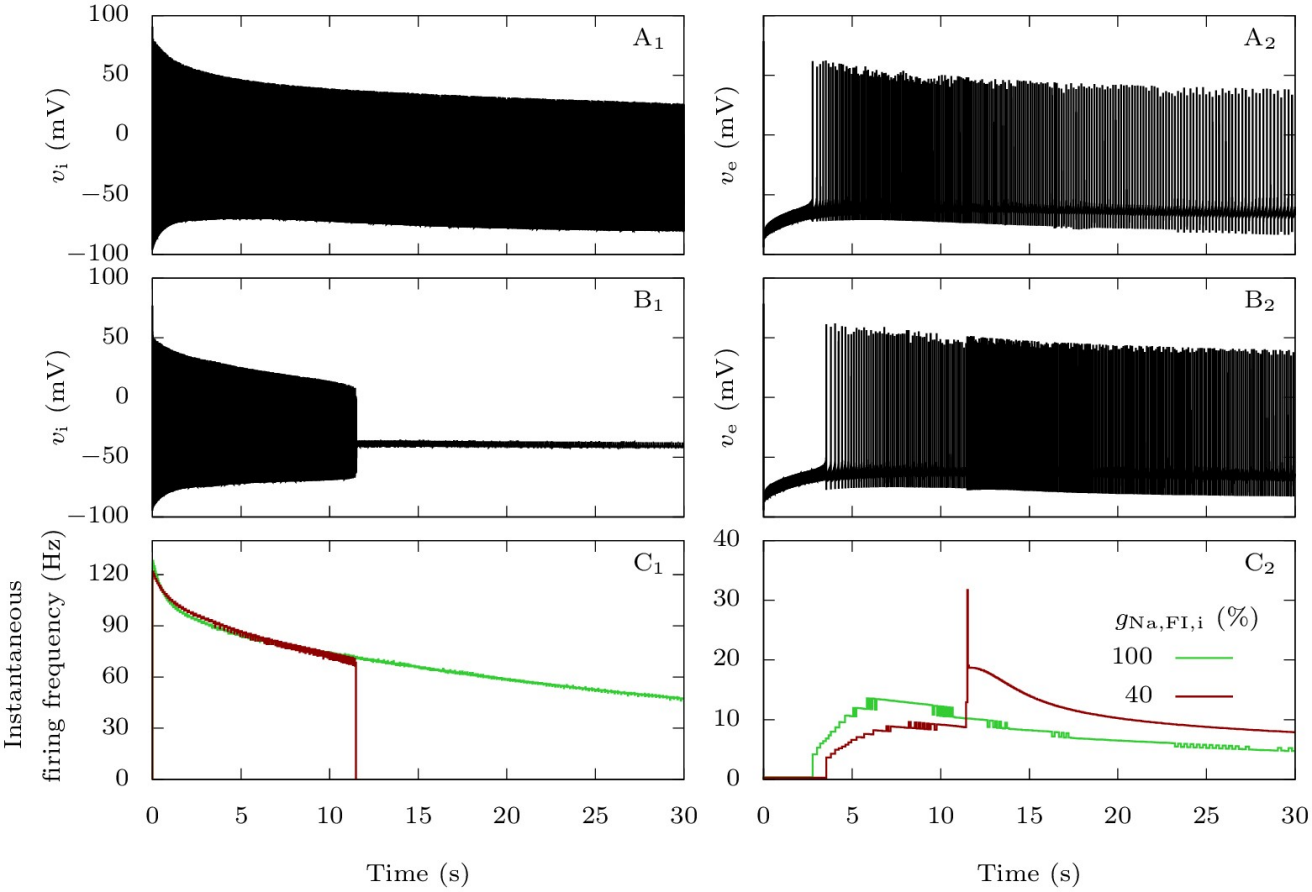
Effect of Na_V_1.1 epileptogenic mutations on the firing frequency of the pyramidal neuron. When both neurons are coupled, taking as initial condition the steady-state when there is no external input, we applied external inputs *g*_D,i_ = *g*_D,e_ = 0.3 mS · cm^−2^. We tested both the default parameters, which represent a wild-type GABAergic neuron, and a sodium fast-inactivating maximal conductance *g*_Na,FI,i_ reduced to 40% of its default value, to model epileptogenic mutations of Na_V_1.1. **A**: Voltage of the GABAergic (**A_1_**) and pyramidal (**A_2_**) neurons in the wild-type condition. **B**: Voltage of the GABAergic (**B_1_**) and pyramidal (**B_2_**) neurons with the epileptogenic mutation. **C**: Instantaneous firing frequency of the GABAergic (**C_1_**) and pyramidal (**C_2_**) neurons in the two conditions.

We saw in the previous section that Na_V_1.1’s epileptogenic loss of function mutations make GABAergic neurons more susceptible to depolarization block. We now focus on the consequences on the firing of the pyramidal neuron in our computational model, when the two neurons are coupled. We stimulated them with strong depolarizing external inputs (*g*_D,i_ = *g*_D,e_ = 0.3 mS · cm^−2^), with or without Na_V_1.1’s epileptogenic loss of function mutations mutation (Fig 12). In the pathological condition, after firing for about ten seconds (Fig 12B_1_ and 12C_1_), the GABAergic neuron enters depolarization block. Simultaneously, we observe a sharp increase of the firing frequency of the pyramidal neuron (Fig 12B_2_ and 12C_2_). This behavior is easily understood: when the GABAergic neuron stops to generate action potentials, the synaptic inhibitory restraint that it exerts on the pyramidal neuron is suddenly removed, allowing the pyramidal neuron to fire at a greater pace. Although this effect cannot be considered epileptiform activity, it may model early hyperexcitability that has been observed in mouse models of Dravet syndrome before the appearance of spontaneous seizures [2, 47].

### 3.3 Comparison of migraine and epilepsy scenarios

**Fig 13:**
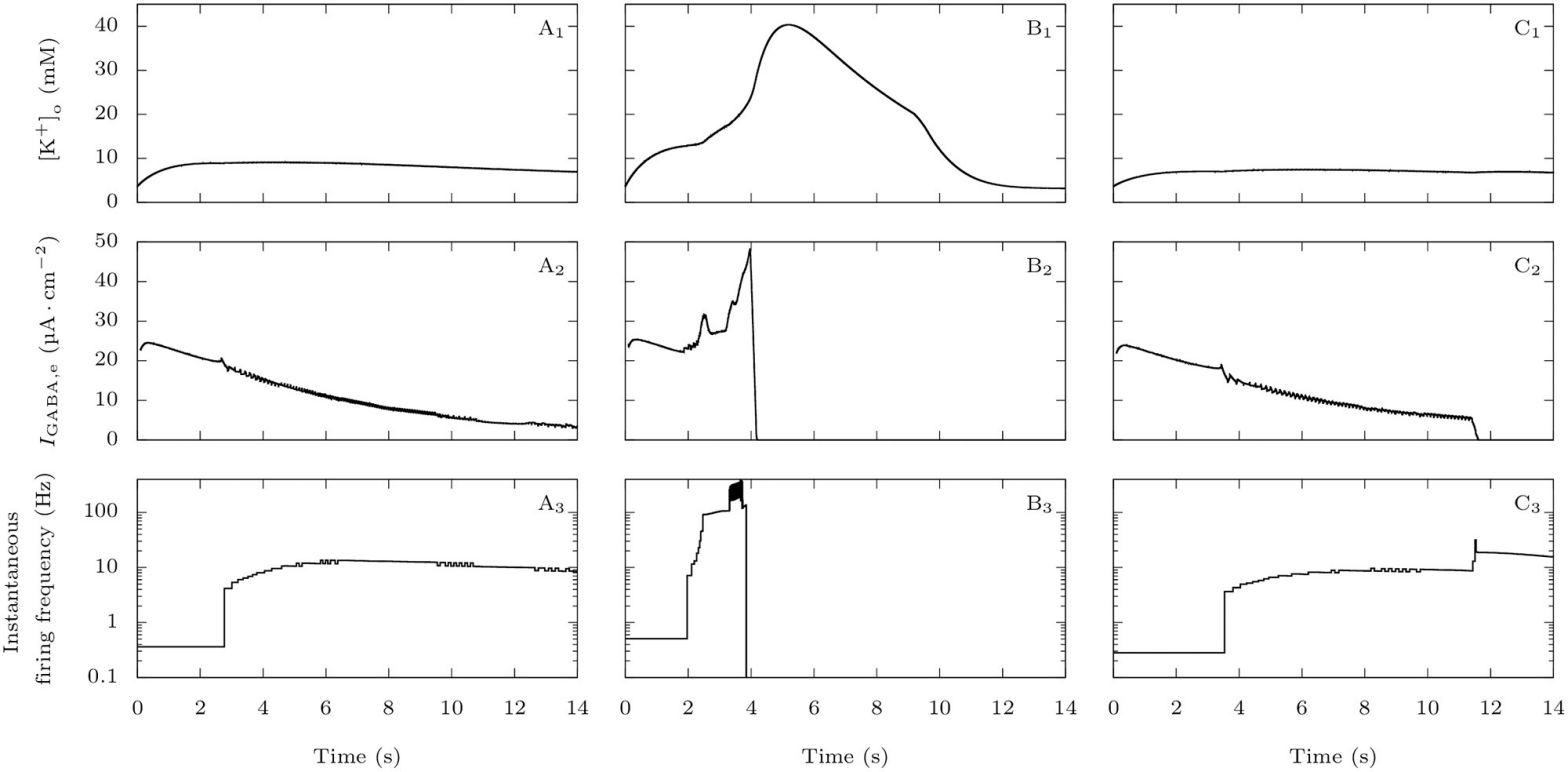
Comparison of migraine and epilepsy scenarios regarding extracellular potassium and GABAergic inhibition. Plots of the extracellular potassium concentration (upper row), GABAergic neuron’s inhibitory current on the pyramidal neuron (second row) and pyramidal neuron’s firing frequency (bottom row) corresponding to the simulations displayed in Figs 4 and 12. For better readability, we averaged the GABAergic currents on a running window of 200 ms. We show the pyramidal neuron’s firing frequency with a logarithmic scale. **A**: Control condition: default parameter values. **B**: Migraine condition: percentage of sodium voltage-gated conductance corresponding to persistent current *p*_Na,P_ = 15%. **C**: Epilepsy condition: sodium fast-inactivating maximal conductance reduced to 40% of its default value.

Fig 13 illustrates the differences, in our model, between the migraine and the epilepsy pathological scenarios. For the representative simulations displayed in Fig 4 and in Fig 12, we compared the evolution of the extracellular potassium concentration, of the inhibitory synaptic current received by the pyramidal neuron, and their effect on the pyramidal neuron’s firing frequency. In the case of the migraine condition (Fig 13B), following an initial accumulation of extracellular potassium a few millimolars larger than in the wild-type condition (Fig 13A), the firing frequency of the pyramidal neuron rises significantly, causing the extracellular potassium to build-up even faster. This happens despite the inhibition from the GABAergic neuron, and it leads to a depolarization block of the pyramidal neuron shortly before four seconds. On the contrary, with the epileptogenic mutation (Fig 13C), the removal of the GABAergic neuron’s inhibitory input on the pyramidal neuron after approximately 11.5 s is responsible for the increase of firing frequency of the pyramidal neuron. The simultaneous increase of extracellular potassium concentration is negligible. Notably, the GABAergic synaptic current never becomes depolarizing (Fig 13A_2_, 13B_2_ and 13C_2_).

## 4 Discussion

We have developed a two-neuron model with one pyramidal neuron and one GABAergic neuron, building upon our previous modeling framework [26]. It captures electrochemical activity leading to either CSD initiation or pro-epileptic hyperexcitability, caused by mutations of the sodium channel Na_V_1.1. It is important to highlight again that our model has been developed to investigate these early events and not fully developed CSD or seizures. For instance, we have not modeled CSD propagation, cell swelling induced by breakdown of ion homeostasis, nor the long lasting depression of activity that can outlasts the neuronal depolarization and may be induced and maintained by other mechanisms [55, 56]. Similarly, we have not modeled the activity of neuronal networks observed during seizures, nor the propagation of seizures [12]. In both cases (CSD initiation and pro-epileptic hyperexcitability), our results suggest the involvement of other mechanisms of network hyperexcitability than the modification of the firing frequency of GABAergic neurons. This is noteworthy, because modifications of firing frequency is one of the principal features investigated in pathologies of neuronal excitability.

### 4.1 FHM-3 mutations

Interestingly, our model did not display a clear-cut increase in firing frequency of the GABAergic neuron implementing a common effect of Na_V_1.1 FHM-3 mutations, although these mutations cause a clear gain of function of the channel. Notably, in a study where FHM-3 mutations were implemented in an extended Hodgkin–Huxley model with dynamic ion concentrations [57], Dahlem et al. reported prolonged action potentials in the mutant model resulting in reduced spiking frequency.

Experimentally, the effect of FHM-3 mutations on firing features is not completely clear yet. An increase of firing frequency has been observed in GABAergic neurons transfected with the FHM-3 mutant L1649Q [7]. Likewise, the application of the toxin Hm1a, which mimics the effect of FHM-3 mutations by enhancing the persistent sodium current, induced an increase of firing frequency in fast spiking cortical GABAergic neurons [29]. However, the same toxin did not modify the firing frequency of CA1 hippocampal GABAergic neurons in [58]. Moreover, comparing heterozygous L1649Q knock-in mice with wild-type littermates, a significant increase of firing frequency has been observed in cortical and hippocampal fast spiking GABAergic neurons, but not in regular spiking cortical and hippocampal GABAergic neurons (Freilinger et al., personal communication; see acknowledgments). The variety of GABAergic neuron subtypes and the great variability of their properties [59, 60] is a possible cause of these discrepancies.

Overall, a noteworthy outcome of the present work is that, in our model, an increase of the firing frequency of the GABAergic neuron is not necessary for FHM-3 mutations to promote network hyperexcitability that leads to CSD initiation. We observed an alternative mechanism in the simulations: although in our model the FHM-3 condition induced just small modifications of the GABAergic neuron’s firing frequency, the ion fluxes at each action potential were increased, leading to a build-up of extracellular potassium. This is possible because each action potential generates larger and more sustained sodium currents that induce increased activation of potassium currents, causing higher net translocation of ions, including potassium, across the membrane, which is consistent with modeling results from Barbieri et al. [61]. This reduces the threshold for CSD initiation and shortens its latency, even in conditions in which the firing frequency of the GABAergic neuron is reduced.

In our previous work [26], we did not directly model Na_V_1.1 FHM-3 mutations. For simplicity, we instead assumed that these gain of function mutations cause hyperactivity of GABAergic neurons. We therefore focused on the effect of an intense firing of the GABAergic neuron on CSD initiation, obtained by increasing the value of the parameter representing a baseline excitatory drive of the GABAergic neuron. Within this framework, we concluded that a high firing frequency of GABAergic neurons can lead to CSD, through extracellular potassium build-up. Here, we improved the model and explicitly modeled FHM-3 mutations with persistent sodium current. We found that the initial accumulation of extracellular potassium leading to the onset of CSD can occur without increase of the GABAergic neuron’s firing frequency (Fig 5), since in our model FHM-3 mutations affect more the ion fluxes at each action potential than the number of action potentials. Our improved model allows us to simulate also the effect of mutations that cause epilepsy.

### 4.2 Epileptogenic mutations

In our model, loss of function of Na_V_1.1, typical of mutations causing epilepsy (including the developmental and epileptic encephalopathy Dravet syndrome), makes GABAergic neurons more susceptible to depolarization block. The action potential frequency during repetitive firing appears unchanged prior to the depolarization block. Simultaneous to the suppression of spike generation by the GABAergic neuron, we observed the transition to a phase of hyperactivity of the pyramidal neuron. This firing pattern cannot be considered as a seizure-like epileptiform activity, but can be interpreted as an earlier stage of hyperexcitability. A limitation of our model is that it only takes into account two neurons, without including any network dynamics. This allowed us to keep its size manageable, but network effects may be necessary for observing seizure-like activity in simulations. Nevertheless, our work suggests the potentially important role of the depolarization block of GABAergic neurons in epilepsies caused by Na_V_1.1 loss of function. In particular, our model could reproduce conditions of the pre-epileptic period identified in mouse models, in which there is network hyperexcitability but not spontaneous seizures [47, 62].

There is experimental evidence in favor of facilitated depolarization block of GABAergic neurons as a mechanism of pro-epileptic network hyperexcitability, for both Na_V_1.1-related and other models.

Our experimental data show that depolarization block is induced by smaller injected currents in fast spiking GABAergic neurons from cortical brain slices of *Scn1a*^+/-^ mice, whereas firing frequency before depolarization block is not significantly modified. There are several papers in which modifications of the initial part of the input-output curve have not been observed in GABAergic neurons of mouse models carrying Na_V_1.1 loss of function mutations. This was the case for instance with dissociated hippocampal neurons [1] and with cortical parvalbumin-positive interneurons in brain slices [63] of *Scn1a*^+/-^ mice, as well as with cortical and hippocampal fast spiking GABAergic neurons of *Scn1a*^RH/+^ mice, which carry the Na_V_1.1 R1648H missense mutation [25]. Notably, the reduced firing frequency observed by numerous studies in the final part of mean input-output relationships, obtained injecting larger depolarizing currents, is likely caused by the earlier depolarization block, which reduces the number of action potentials elicited by large depolarizations in neurons expressing Na_V_1.1 loss of function mutants. In fact, the most consistent effect observed in the representative fast spiking discharges displayed by most of the papers is earlier depolarization block. It should be highlighted again that these traces have not been included in the mean input-output curves that we have presented here.

In an experimental model in which seizure-like events were induced in rat hippocampal slices from wild type mice using the potassium channel blocker 4-aminopyridine together with decreased magnesium, a sequence of events similar to what we obtained in our simulations was reported: seizure generation correlated with long-lasting depolarization blocks in GABAergic neurons and the simultaneous increase of firing frequency in pyramidal cells [64]. Another study, which used 4-aminopyridine together with decreased magnesium and local applications of NMDA to focally induce epileptiform ictal activity in wild type cortical rodent brain slices, suggested that depolarization block of fast spiking GABAergic neurons allows the recruitment of clusters of pyramidal cells into propagating epileptiform discharges [65]. Consistently, a computational modeling study found that seizure-like activity can arise as the result of depolarization block of inhibitory neurons and investigated possible bifurcation structures for this transition [66].

Interestingly, a neuronal mass computational model of Dravet syndrome generated, when abnormal depolarizing GABA_A_ currents were implemented (which would make GABAergic synaptic connections excitatory), seizure-like activity that was similar to some EEG patterns observed in Dravet syndrome patients [67]. The rationale for implementing this effect was a hypothetical remodeling in which the initial Na_V_1.1-induced hyperexcitability leads to the cleavage of KCC2 co-transporters, resulting in intracellular accumulation of chloride in pyramidal neurons. Our model takes into account KCC2 co-transporters and dynamic chloride concentrations, but we did not implement remodeling that leads to reduced KCC2 function. In fact, although depolarizing GABA has been reported in a mouse model of Dravet syndrome, it was not found to be significantly involved in seizure activity [68]. Conversely, it would be interesting to include depolarization block of GABAergic neurons in the neuronal mass computational model of Dravet syndrome [67], for example by adapting the corresponding wave-to-pulse function for taking it into account.

### 4.3 Conclusions and perspectives

Overall, our results suggest that depolarization block can be involved in the mechanism of both gain of function migraine mutations and loss of function epilepsy mutations of Na_V_1.1, but with different features. In the migraine condition spiking-induced increased extracellular potassium leads to depolarization block of both GABAergic and glutamatergic neurons, whereas in the epilepsy condition depolarization block of GABAergic neurons leads to hyperexcitability of glutamatergic neurons. Notably, modifications of firing frequency of the GABAergic neurons are not necessary for inducing these effects.

Our results that disclose different pathological mechanisms leading to CSD and epileptic activity are consistent with the finding that often epileptic networks are resistant to CSD induction: In several models, the propensity to CSD generation seems to decline during the course of epileptogenesis, whereas the propensity to spontaneous epileptic seizures increases. For instance, the threshold for high potassium-induced CSD was increased in neocortical slices both from patients who had undergone surgery for intractable epilepsy and from chronic epilepsy rats following pilocarpine-induced status epilepticus, whereas brain slices from age-matched healthy control rats that showed a lower threshold [69]. Similarly, the propensity to spreading depolarization was reduced during the epileptogenesis induced by blood-brain barrier disruption and pentylenetetrazol kindling in rats [70, 71]. Thus, the dissociation between propensity to spreading depolarization on the one hand and epileptic seizures on the other hand could be a general feature beyond FHM3 and *SCN1A*-linked epilepsies.

A further future investigation would be to develop a reduced model more amenable to theoretical analysis while retaining the salient features of the present model. Bifurcation theory is indeed a very powerful tool to dissect the spectrum of activity regimes that a model can produce, as well as offer a cartography of these regimes in parameter space. Furthermore, the obvious presence of multiple timescales brings a strategy to reduce the model. We initiated this approach in the present work by studying a particular fast subsystem of the full model in Fig 11. However, the dimension of the current model makes it prohibitive to perform a thorough bifurcation analysis as well as to fully exploit its multi-timescale structure. Therefore, a future objective will be to build up a simpler model, keeping the main features of this detailed model, which will allow a more in-depth analysis. In particular, we plan to study bifurcation scenarios associated with the transitions to migraine and epileptiform activity in a minimal bio-inspired slow-fast model, with ion concentrations as slow processes driving the system, via threshold effects underpinning these transitions to pathological activity using tools from multiple-timescale analysis.
